# The IkappaB Kinase Family Phosphorylates the Parkinson’s Disease Kinase LRRK2 at Ser935 and Ser910 during Toll-Like Receptor Signaling

**DOI:** 10.1371/journal.pone.0039132

**Published:** 2012-06-18

**Authors:** Nicolas Dzamko, Francisco Inesta-Vaquera, Jiazhen Zhang, Chengsong Xie, Huaibin Cai, Simon Arthur, Li Tan, Hwanguen Choi, Nathanael Gray, Philip Cohen, Patrick Pedrioli, Kristopher Clark, Dario R. Alessi

**Affiliations:** 1 MRC Protein Phosphorylation Unit, College of Life Sciences, University of Dundee, Dow Street, Dundee, Scotland; 2 Transgenic Section, Laboratory of Neurogenetics, National Institute of Aging, National Institute of Mental Health, National Institutes of Health, Bethesda, Maryland, United States of America; 3 Department of Cancer Biology, Dana-Farber Cancer Institute, Harvard Medical School, Boston, Massachusetts, United States of America; 4 Department of Biological Chemistry and Molecular Pharmacology, Dana-Farber Cancer Institute, Harvard Medical School, Boston, Massachusetts, United States of America; 5 Scottish Institute of Life Sciences, College of Life Sciences, University of Dundee, Dow Street, Dundee Scotland; Johns Hopkins, United States of America

## Abstract

Mutations in leucine-rich repeat kinase 2 (LRRK2) are strongly associated with late-onset autosomal dominant Parkinson's disease. LRRK2 is highly expressed in immune cells and recent work points towards a link between LRRK2 and innate immunity. Here we demonstrate that stimulation of the Toll-Like Receptor (TLR) pathway by MyD88-dependent agonists in bone marrow-derived macrophages (BMDMs) or RAW264.7 macrophages induces marked phosphorylation of LRRK2 at Ser910 and Ser935, the phosphorylation sites that regulate the binding of 14-3-3 to LRRK2. Phosphorylation of these residues is prevented by knock-out of MyD88 in BMDMs, but not the alternative TLR adaptor protein TRIF. Utilising both pharmacological inhibitors, including a new TAK1 inhibitor, NG25, and genetic models, we provide evidence that both the canonical (IKKα and IKKβ) and IKK-related (IKKε and TBK1) kinases mediate TLR agonist induced phosphorylation of LRRK2 in vivo. Moreover, all four IKK members directly phosphorylate LRRK2 at Ser910 and Ser935 in vitro. Consistent with previous work describing Ser910 and Ser935 as pharmacodynamic biomarkers of LRRK2 activity, we find that the TLR independent basal phosphorylation of LRRK2 at Ser910 and Ser935 is abolished following treatment of macrophages with LRRK2 kinase inhibitors. However, the increased phosphorylation of Ser910 and Ser935 induced by activation of the MyD88 pathway is insensitive to LRRK2 kinase inhibitors. Finally, employing LRRK2-deficient BMDMs, we present data indicating that LRRK2 does not play a major role in regulating the secretion of inflammatory cytokines induced by activation of the MyD88 pathway. Our findings provide the first direct link between LRRK2 and the IKKs that mediate many immune responses. Further work is required to uncover the physiological roles that phosphorylation of LRRK2 by IKKs play in controlling macrophage biology and to determine how phosphorylation of LRRK2 by IKKs impacts upon the use of Ser910 and Ser935 as pharmacodynamic biomarkers.

## Introduction

Mutations in the gene encoding the protein kinase LRRK2 (leucine rich repeat kinase 2) cause autosomal dominant Parkinson’s disease [1], [2]. LRRK2 is a large multi-domain protein kinase (2527 residues), consisting of leucine-rich repeats (residues 983–1320), a GTPase domain (residues 1335–1504), a COR [C-terminal of Roc (Ras in complex proteins)] domain (residues 1517–1843), a serine/threonine protein kinase domain (residues 1875–2132) and a WD40 repeat (residues 2101–2517) [3], [4]. Over 40 mutations in LRRK2 have been reported thus far which mainly comprise amino acid substitutions [5]. The most common mutation replaces glycine 2019 with a serine within the magnesium-binding DYG motif (DFG motif in most other kinases) of the kinase domain, thereby increasing LRRK2 activity 3-fold [6], [7]. This indicates that inhibitors of LRRK2 activity might be of therapeutic benefit for the treatment of Parkinson’s disease. Little is known about the physiological role of LRRK2 and to date no properly validated substrates have been identified.

Recent work has revealed that LRRK2 interacts with 14-3-3 phospho-binding adaptor isoforms [8]. Phosphorylation of Ser910 and Ser935 located prior to the leucine rich repeat domain mediates binding of 14-3-3 isoforms to LRRK2 [8]. This binding may be linked to Parkinson’s disease as phosphorylation of Ser910 and Ser935 and 14-3-3 binding is inhibited by five of the six validated LRRK2 pathogenic mutations (R1441C, R1441G, R1441H, Y1699C and I2020T) [8], [9]. Intriguingly, treatment of cell and animal models with structurally unrelated LRRK2 kinase inhibitors also results in dephosphorylation of Ser910 and Ser935, again accompanied by loss of 14-3-3 binding [10], [11]. Recent work has also revealed that LRRK2 inhibitors induce the dephosphorylation of two other nearby residues that do not control 14-3-3 binding (Ser955 and Ser973) [12]. The kinase(s) and phosphatase(s) that act on Ser910, Ser935, Ser955 and Ser973 are unknown, but the evidence suggests that these residues are not phosphorylated by LRRK2 itself [10].

Recent studies have demonstrated that LRRK2 is highly expressed in immune cells, in particular, macrophages and B-lymphocytes [13]–[15]. Furthermore the expression of LRRK2 is reportedly increased in macrophages stimulated with interferon gamma (IFNγ)[13]. Another recent study has also suggested that LRRK2 can modulate inflammatory cytokine secretion by promoting cytoplasmic localisation of the Nuclear Factor of Activated T-cells (NFAT) transcription factor, through a mechanism that does not involve regulation of NFAT phosphorylation [16].

Innate immune signaling can be triggered by activation of Toll-like receptors (TLRs). These receptors consist of a family of membrane proteins that sense pathogen-associated molecular patterns [17], [18]. TLR’s 1,2,5,6,7,8 and 9 signal through the adaptor protein Myeloid Differentiation primary response gene-88 (MyD88) [19], [20]. Stimulation of the MyD88 dependent pathway results in the TNF receptor-associated factor 6 (TRAF6)-dependent activation of the Transforming Growth Factor β–activated kinase 1 (TAK1), which subsequently phosphorylates and activates the “canonical” IκB kinases (termed IKKα and IKKβ) to induce transcription of Nuclear Factor-KappaB (NFκB) dependent genes such as Interleukin-6 (IL-6) and Tumour Necrosis Factor-alpha (TNFα) [21]. Activation of the MyD88 pathway also results in activation of the “non-canonical” IKK related kinases termed TANK-binding kinase 1 (TBK1) and IKKε, which act to limit the activation of the canonical IKKs [22], [23]. TLR3 signals through an alternative adaptor protein termed TIR-domain-containing adapter-inducing Interferon-β (TRIF) [24]. This leads to the activation of TBK1 and IKKε, which phosphorylate Interferon Regulatory Factor-3 (IRF3) leading to induction of interferon-β (IFNβ) [17]. TLR4, which is activated by lipopolysaccharide (LPS), is unique as it signals through both the MyD88 and TRIF pathways [25]. Interestingly, although both the TRIF and MyD88 signalling pathways lead to activation of IKKε/TBK1, only activation of the TRIF pathway is capable of inducing the phosphorylation of IRF3 illustrating that biological consequences of TBK1/IKKε activation depends on whether these enzymes are activated by the MyD88 or TRIF pathways [23].

In this study we have raised highly sensitive rabbit monoclonal antibodies that enable detection of the LRRK2 protein and its phosphorylation at Ser935 by immunoblot analysis of cell extracts of primary bone marrow-derived macrophages (BMDMs). We utilised these antibodies to determine whether LRRK2 protein expression or phosphorylation were altered in macrophages treated with a number of agonists of immune signalling pathways. Strikingly, we find that activation of the MyD88-dependent pathway by TLR agonists induces marked phosphorylation of LRRK2 at Ser935. We present biochemical, pharmacological as well as genetic evidence that TBK1 and IKKε, as well as IKKα and IKKβ, mediate phosphorylation of Ser935 as well as Ser910 and Ser955 following activation of the MyD88-dependent TLR pathway. The present study identifies LRRK2 as a novel physiological substrate of IKKs and provides further intriguing evidence supporting the view that LRRK2 may play a role in immune signaling.

## Methods

### Reagents and General Methods

Toll receptor agonists were purchased from Invivogen. Signal transduction inhibitors of TBK1/IKKε (MRT67307) [22], TAK1 (5Z-7-oxozeaenol) [26], IKKβ (BI605906) [22] and LRRK2 (LRRK2-IN1 [11] and CZC25146 [27]) were provided by the Division of Signal Transduction Therapy (DSTT), University of Dundee, Scotland. A structurally unrelated TAK1 inhibitor, termed NG25, was provided by Dr Nathanael Gray (see Method S1 for the chemical synthesis of NG25). Tissue culture reagents were from Life Technologies. Restriction digests, DNA ligations and other recombinant DNA procedures were performed using standard protocols. DNA mutagenesis was performed using the Quickchange site directed mutagenesis kit (Stratagene). DNA constructs used for transfection were purified from DH5α *E*.*Coli* using Qiagen plasmid maxi-prep kits. All DNA constructs were verified by sequencing performed by the sequencing service, School of Life Sciences, University of Dundee. The following substrates were generated by the DSTT by expression in BL21 *E*.*Coli* with an N-terminal GST tag (IκBα 2-54, LRRK2 882-1300, IRF3 1-427 or maltose binding protein (MBP) tag (MKK6 2-334) and purified using glutathione Sepharose or amylose resin respectively. The kinases IKKε (1-716) and TAK1 (1-103)/TAB1 (437-504) fusion were expressed in baculovirus with N-terminal 6× His tags and purified using Ni^2+^-NTA agarose.

### Antibodies

Antigens used to generate total LRRK2, LRRK2 phosphorylated at Ser910 and LRRK2 phosphorylated at Ser935 were the same as used to generate the sheep polyclonal antibodies that have been described previously [28] [8]. Rabbit monoclonal antibodies were generated in conjunction with Epitomics. 211 clones and subclones were screened by immunoblot using both recombinant and endogenous LRRK2 to select the most sensitive. The optimal clones were expanded for the production of purified antibody by Epitomics. These antibodies are now distributed by the Michael J Fox Foundation and can be purchased from Epitomics (Total LRRK2[100-500] antibody catalogue number 5097-1; Phospho Ser935 LRRK2 catalogue number 5099-1 and Phospho Ser910 antibody that detects only human but not mouse LRRK2 catalogue number is 5098-1. For immunoblot of mouse LRRK2, a Phospho Ser910 sheep polyclonal antibody was used. All other antibodies employed in this study were purchased from Cell Signaling Technology.

### Animals

Mice were maintained in accordance with UK and EU regulations, and work was covered by an appropriate home office license (60/3811) which was subject to review by the University of Dundee Ethical Review Committee. LRRK2 knock-out mice were generated and provided by Dr Huaibin Cai, Transgenics Section, NIH, USA and have been described previously [29]. MyD88 [30] and TRIF [24] null mice were generated and provided by Dr Shizuo Akira, Department of Host Defense, Osaka University, Japan. Bone marrow from IKKα double Ser176A and Ser180A knock-in mice [31] was generously provided by Dr Toby Lawrence, Inflammation Biology Group, Centre d’Immunologie, Marseille-Luminy, France.

### Cell Culture, Treatments and Lysis

L929 cells were maintained in RPMI +10% (v/v) heat inactivated FBS, 2 mM L-glutamine and 1× penicillin/streptomycin. To generate L929 conditioned media, cells were seeded at 2×10^5^ cells per ml and media collected 7 days and 14 days after seeding. Media was filtered through a 0.4 µM filter and stored at −80°C. 50 ml of Day 7 and 50 ml of Day 14 L929 conditioned media was added to 400 ml RPMI containing 10% FBS to create a base media for differentiating bone marrow derived macrophages [32]. Bone marrow precursor cells were extracted from mouse femurs as described previously [22]. Briefly, femurs were extracted under sterile conditions and bone marrow flushed with PBS. Cells were washed with PBS and resuspended in L929 conditioned media for plating onto non-tissue culture treated petri dishes. Half of the media was changed after 4 days. After 7 days adherent macrophages were scraped from the petri dishes with versene and then plated into tissue culture grade plastic 12 well, 6 well or 10 cm dishes and left for 24 h to re-attach before experiments. Macrophage cultures were routinely >95% pure as identified by flow cytometry analysis of the macrophage surface marker F4/80. RAW264.7 cells were grown in DMEM containing 10% (v/v) heat inactivated FBS and 1× penicilin/streptomycin. HEK293 cells were grown in DMEM containing 10% (v/v) FBS and 1× penicillin/streptomycin. HEK293 cells were transfected using the PEI method. TLR agonists were dissolved in LAL water (Invivogen) and inhibitors dissolved in sterile DMSO. An equivalent volume of DMSO was added to control samples where appropriate. Following treatment, cells were lysed in buffer containing 50 mM Tris.HCL pH 7.5, 1 mM EGTA, 1 mM EDTA, 1 mM sodium orthovanadate, 10 mM β-glycerophosphate, 50 mM NAF, 5 mM sodium pyrophosphate, 0.27 M sucrose, 0.1% (v/v) 2-mercaptoethanol and 1% (v/v) Triton X-100. Lysates were centrifuged at 12000 rpm for 20 min and supernatants retained for protein assay (Coomassie protein assay reagent, Thermo Scientific) and immunoblot.

### Immunoblot Analysis

Cell lysates (8–20 µg) were resolved by SDS-PAGE using either 8%, 10% or Novex 4–12% gradient gels (Invitrogen) and transferred to nitrocellulose membranes in the presence of 15% (v/v) methanol. Membranes were blocked with 5% (w/v) skimmed milk powder in TBST (Tris-buffered saline with Tween 20) buffer (50 mM Tris.HCl pH 7.5, 0.15 M NaCl and 0.1%(v/v) Tween 20). Rabbit monoclonal LRRK2 antibodies were used at a concentration of 100 ng/ml diluted in TBST with 5% (w/v) bovine serum albumin (BSA). Cell Signaling Technology antibodies were used at a dilution of 1/1000 overnight in TBST with 5% (w/v) BSA. Anti-rabbit HRP (horseradish peroxidase) secondary antibodies (Sigma) were used to detect immune complexes using enhanced chemiluminescence reagent. 14-3-3 overlay far western analysis was performed as described previously [8].

### LRRK2 Peptide Kinase Assays

Kinase assays were performed using endogenous LRRK2 immunoprecipitated from 3 mg RAW264.7 cell lysate. Assays were set up in a volume of 50 µl containing 50 mM Tris/HCl, pH 7.5, 0.1 mM EGTA, 1 mM DTT, 10 mM MgCl_2_ 0.1 mM [γ-^32^P]ATP (300 cpm/pmol) and 20 µM Nictide peptide substrate. Reactions were terminated after 20 min at 30°C by applying 40 µl of the reaction mixture to P81 phosphocellulose paper and immersion in 50 mM phosphoric acid. After washing, reaction products were quantified by Cerenkov counting. Kinase activity is reported as pmol of ATP incorporated into Nictide per minute per mg of lystate immunoprecipitated from as previously described [28].

### SILAC Phosphoproteomics

RAW264.7 cells were labelled using the Stable Isotope Labelling of Amino Acids in Cell Culture (SILAC) method. Cells were treated with 2 µM MRT67307 or vehicle control for 1 h and subsequently, left unstimulated or stimulated with 1 µg/ml Pam_3_CSK_4_ for 30 min. The cells were lysed, proteins were reduced, alkylated and digested with trypsin. Phosphopeptides were enriched by sequential hydrophilic (HILIC) chromatography followed by Fe^3+^-IMAC chromatography [33]. Phosphopeptides were measured by LC-MS/MS on Thermo Fisher Scientific LTQ Orbitrap Velos instrument set to perform top-15 data-dependent CID analysis in the 350–1600 m/z range using a resolution of 60000 for the precursor scan and a minimal intensity for sequencing of 10000 counts. Monoisotopic precursor selection was used and +1 as well as unassigned charge states were excluded from sequencing. Dynamic exclusion was set to a repeat count of 2 within 30 sec, with exclusion duration of 90 sec and an exclusion mass width of 10 ppm. The data was analysed using MaxQuant [34].

### qRT-PCR

RNA was extracted using Rneasy micro kits (Qiagen) with QIAshredders (Qiagen) used for cell disruption. RNA was reverse transcribed using the IQscript system (Biorad). qRT-PCR was performed on a Biorad CFX realtime system using 20 µl reactions in duplicate. Ssofast Evergreen Supermix (Biorad) was used to measure amplification of diluted cDNA, which was then normalized to the housekeeping gene GAPDH. Fold difference in gene expression was calculated using the comparative Ct method (2^−ddct^). PCR efficiencies were similar between primer sets and the expression of the housekeeping gene did not change with TLR agonist treatment. Primer sets for inflammatory cytokine genes are listed in Table S4.

### Cytokine Measurements

For the measurement of inflammatory cytokine release in culture media, BMDMs were plated at a density of 1×10^6^ in 6 well tissue culture plates. Media was collected 8 h post stimulation and stored at −80°C until analysis. TNFα, IL-6, IL-12(p40), RANTES, IL-10, IL-1β, MCP-1 and KC were measured using a bioplex-pro cytokine assay and Luminex technology (Biorad) according to the manufacturers instructions.

### In vitro Phosphorylation Reactions

In vitro phosphorylation reactions were performed in a volume of 50 µl containing 50 mM Tris.HCl, pH 7.5, 0.1 mM EGTA, 1 mM DTT, 10 mM MgCl_2_, 0.1 mM [γ-^32^P]ATP (300 cpm/pmol), 2 µM substrate and kinase. For baculovirus expressed and purified kinase 0.2 µg of recombinant kinase was used per reaction. For kinases expressed in HEK293 cells, 5 µl of HA-agarose was used to immunoprecipitate the overexpressed kinase from 1–5 mg of lysate. Beads were washed five times with lysis buffer containing 0.5 M NaCl and once in kinase assay buffer before being utilised for in vitro phosphorylation reactions. Assays were undertaken at 30°C for 30 min and terminated by addition of 4× lithium dodecyl sulfate (LDS) sample buffer (Invitrogen).

## Results

### Generation of Rabbit Monoclonal Antibodies

**Figure 1:**
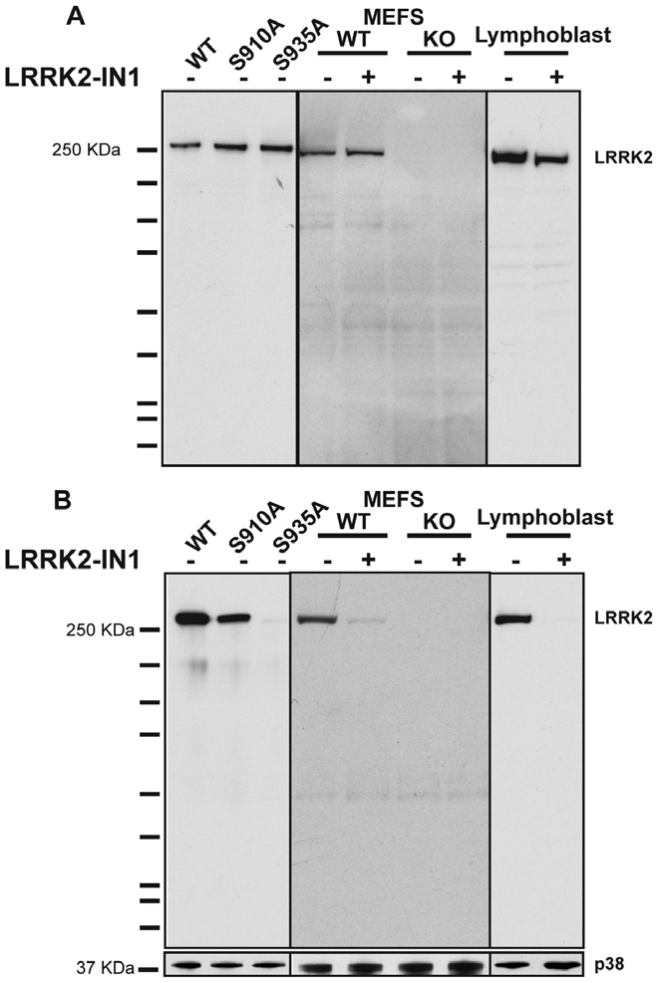
Generation of rabbit monoclonal LRRK2 antibodies. (**A**) Purified rabbit monoclonal antibody raised against residues 100–500 of human LRRK2 was used to immunoblot 5 µg HEK293 cell lysate containing overexpressed GFP-LRRK2 variants, 30 µg LRRK2 wild type and knock-out mouse embryonic fibroblast (MEF) lysate and 30 µg human lymphoblastoid cell lysate. Lymphoblast and MEF cells were treated plus or minus 1 µM LRRK2-IN1 for 2 h. A longer exposure was required for the MEF LRRK2 signal. (**B**) As in **A** except a rabbit monoclonal antibody to LRRK2 phosphorylated at Ser935 was used.

We generated rabbit monoclonal antibodies that efficiently recognize endogenous LRRK2 protein and LRRK2 phosphorylated at Ser935 in cell extracts. These antibodies recognise LRRK2 as the major band on immunoblot analysis and their specificity is confirmed by loss of signal in LRRK2 knock-out fibroblasts (Fig 1 A & Fig 1B). Moreover, mutation of Ser935 to Ala or treatment with the LRRK2-IN1 inhibitor to induce dephosphorylation of LRRK2, abolished recognition by the LRRK2 Ser935 phospho-antibody (Fig 1B).

### TLR Activation Stimulates LRRK2 Ser935 Phosphorylation

**Figure 2:**
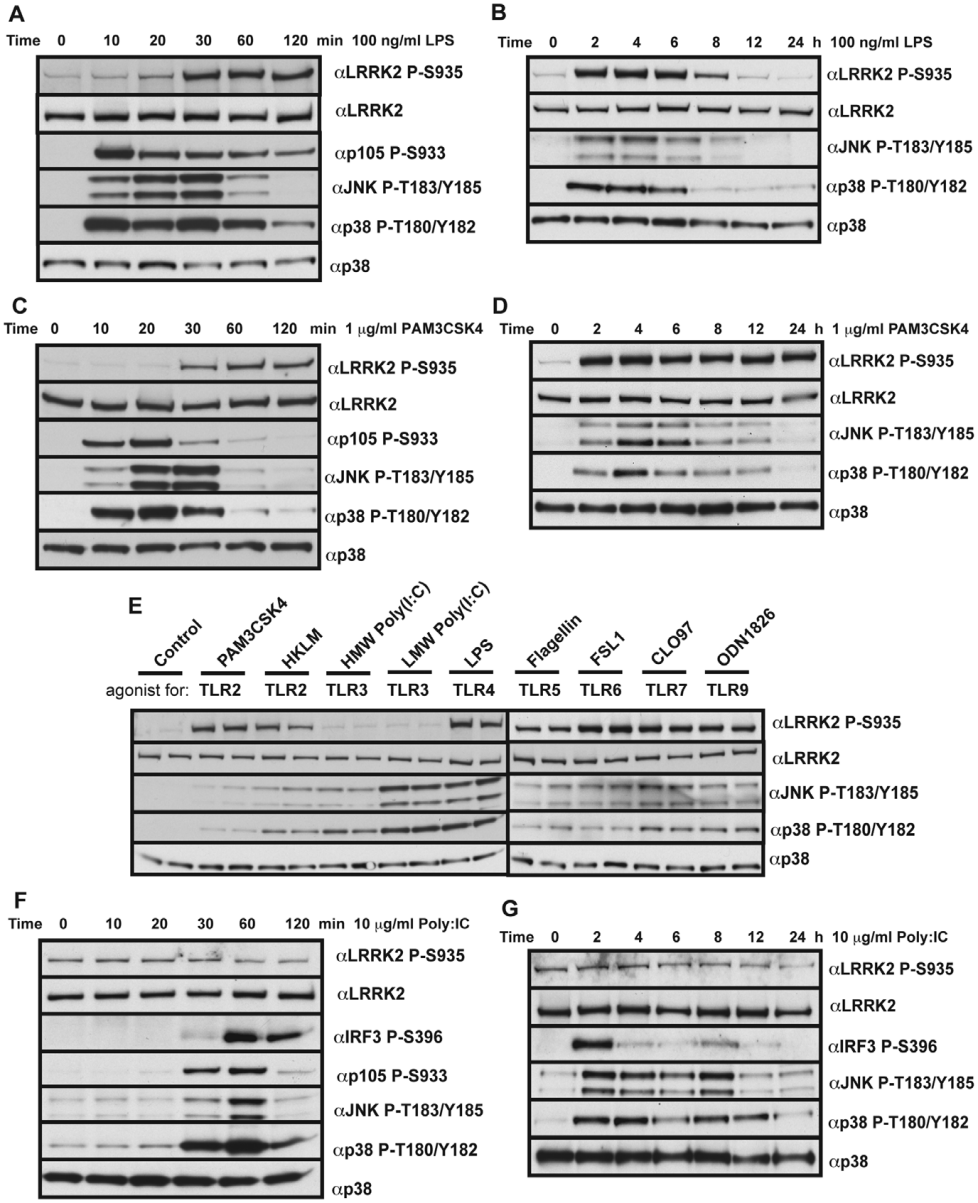
TLR agonists increase LRRK2 phosphorylation at Ser935. (**A & B**)**.** Primary bone marrow derived macrophages were treated with 100 ng/ml LPS for the indicated time points before cell lysis and immunoblot with indicated antibodies. (**C & D**) Primary bone marrow derived macrophages were treated with 1 µg/ml Pam_3_CSK_4_ for the indicated time points before cell lysis and immunoblot with indicated antibodies. (**E**) Primary bone marrow derived macrophages were treated with the following TLR agonists for 1 h. 1 µg/ml Pam_3_CSK_4_, 10^8^ cells HKLM, 10 µg/ml LMW and HMW Poly(I:C), 100 ng/ml LPS, 10 µg/ml Flagellin, 1 µg/ml FSL1, 1 µM CLO97 and 2.5 µM ODN1826. (**F & G**) Primary bone marrow derived macrophages were treated with 10 µg/ml Poly(I:C) for the indicated time points before cell lysis and immunoblot with indicated antibodies. All immunoblots are representative of at least two independent experiments.

It has recently been described that the JAK-STAT pathway agonist IFNγ can induce the expression of LRRK2 [13]. We therefore utilised our newly generated antibodies to determine if other agonists of immune signalling pathways altered LRRK2 expression or phosphorylation in macrophages. We first examined levels of LRRK2 protein as well as Ser935 phosphorylation following stimulation of BMDMs with LPS, an agonist for TLR4, over a 24 h period (Fig 2A & 2B). Although no marked changes in LRRK2 protein expression were observed, LPS induced a striking phosphorylation of Ser935 within 30 min (Fig 2A). This was sustained for up to 6 h and gradually decreased to basal levels by 12 h (Fig 2B). In parallel the phosphorylation of p105 at Ser933 mediated by IKKα/IKKβ and phosphorylation of the JNK and p38 MAP kinases increased within 10 min in response to LPS and declined subsequently, as expected (Fig 2A and 2B).

We next treated BMDMs with the bacterial lipopeptide Pam3-Cys-Ser-Lys4 (Pam_3_CSK_4_), an agonist that stimulates signalling through the MyD88 pathway through the heterodimeric TLR1/TLR2 complex. This similarly induced phosphorylation of LRRK2 at Ser935 within 30 min, again slower than the phosphorylation of p105, JNK and p38 (Fig 2C). Phosphorylation of LRRK2 at Ser935 induced by Pam_3_CSK_4_ was sustained for 24 h while the peak phosphorylation of p105, JNK and p38 was more transient (Fig 2D).

We next stimulated BMDMs with a panel of TLR agonists revealing that all agonists tested that signal via the MYD88 pathway namely TLR2 (Heat Killed Listeria monocytogenes HKLM), TLR5 (Flagellin), TLR6 (Synthetic diacylated lipoprotein, FSL1), TLR7 (Imidazoquinoline compound-97, CLO97) and TLR9 (synthetic oligodeoxynucleotide containing unmethylated CpG motifs termed ODN1826) induced phosphorylation of LRRK2 at Ser935 similarly to LPS and Pam_3_CSK_4_ (Fig 2E). In contrast, TLR3 agonists (high and low molecular weight Polyinosine-polycytidylic acid, PolyI:C) that signal through the TRIF pathway, failed to induce Ser935 phosphorylation. We also tested TLR3 agonists over a 24 h time period and observed no significant phosphorylation of Ser935 despite observing phosphorylation of p105, p38, JNK and IRF3 indicating that activation of the TRIF pathway had occurred (Fig 2F & 2G). We also investigated the effect of TLR agonists on LRRK2 phosphorylation in RAW264.7 macrophage cells. In this cell line LRRK2 Ser935 phosphorylation was also increased by Pam_3_CSK_4_, LPS, FSL1 and ODN1826 (Fig S1). As LRRK2 is highly abundant in RAW264.7 cells, we were also able to detect an increase in the phosphorylation of Ser910 (Fig S1).

**Figure 3:**
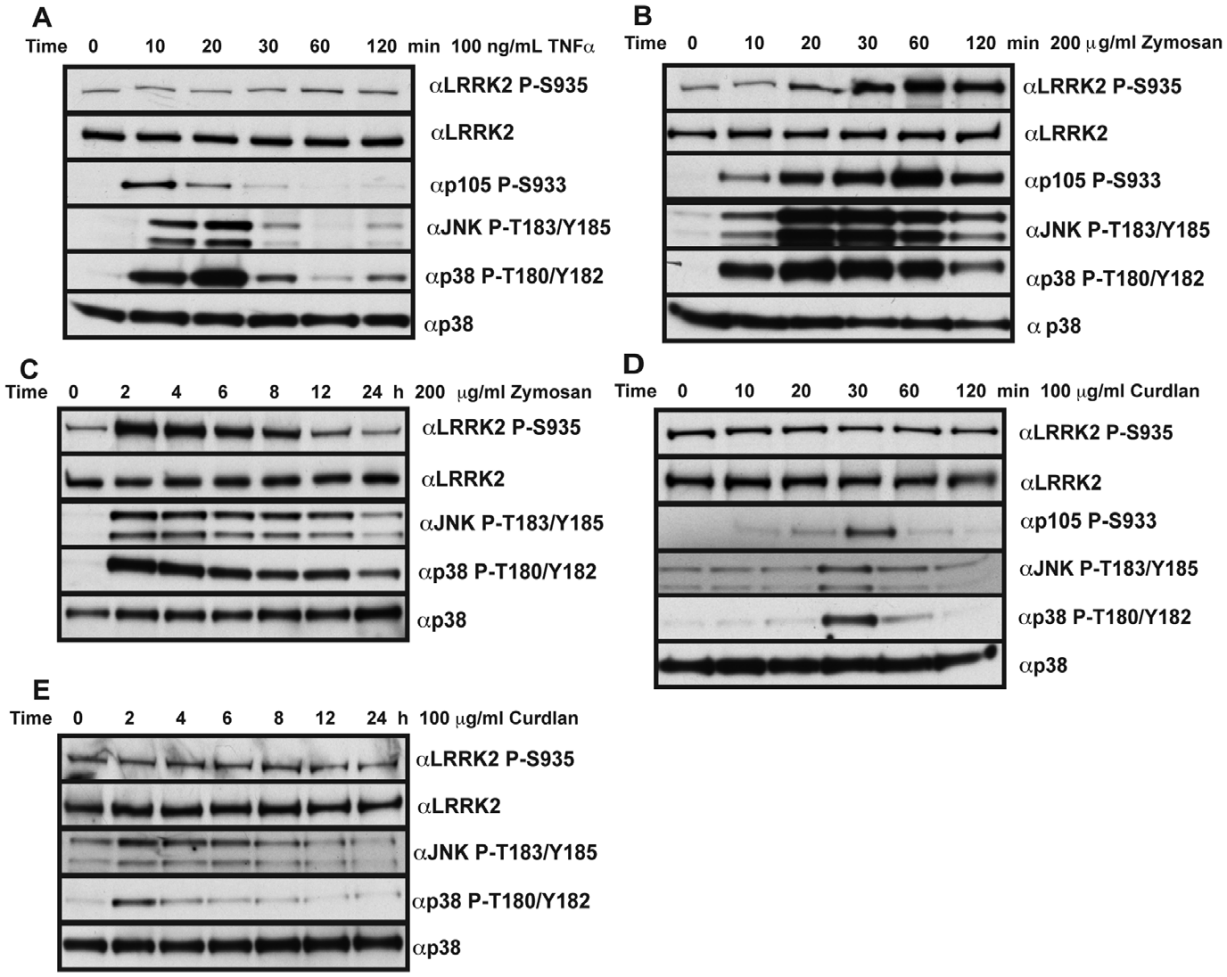
Non-TLR immune agonists fail to increase LRRK2 phosphorylation. (**A**) Primary bone marrow derived macrophages were treated with 100 ng/ml TNFα for the indicated time points before cell lysis and immunoblot with the indicated antibodies. (**B & C**) As in **A** except 200 µg/ml zymosan was used. (**D and E**) as in **A** except that 100 µg/ml curdlan was used. Immunoblots are representative of at least two independent experiments.

We also stimulated BMDMs with agonists of other immune signaling pathways including TNFα (Fig 3A), the Nucleotide-binding oligomerisation domain-containing protein 2 (NOD2) agonist muramyl dipeptide (MDP) and the NOD1 agonist ie-DAP (data not shown). We observed that these failed to induce phosphorylation of LRRK2 at Ser935 over a 2h time course, despite inducing phosphorylation of p105 and p38 (TNFα & NOD2) and JNK (TNFα). In contrast, the yeast cell wall glucan component zymosan that activates both TLR2 and the C-type lectin receptor dectin-1, promoted Ser935 phosphorylation of LRRK2 (Fig 3B). The time course of Ser935 phosphorylation following zymosan treatment followed that of LPS with phosphorylation markedly increased by 30 min (Fig 3B) and remaining elevated for 8h (Fig 3C). The effect of zymosan in inducing Ser935 phosphorylation is likely to be mediated via the TLR2 pathway however, as the dectin-1 specific agonist curdlan failed to induce phosphorylation of Ser935 over the same time course (Fig 3D and 3E).

### LRRK2 Phosphorylation is MyD88-dependent

**Figure 4:**
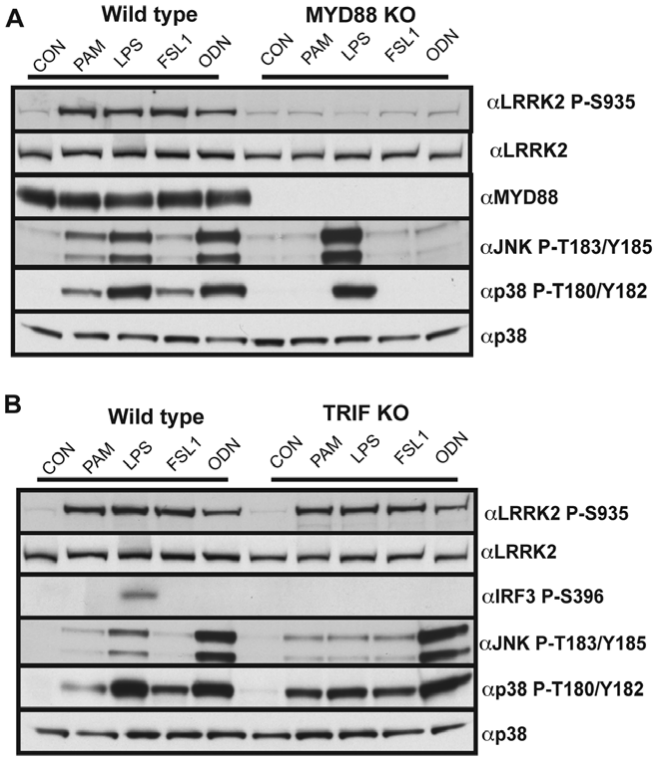
TLR induced LRRK2 Ser935 phosphorylation is MYD88-dependent. (**A**) Primary bone marrow derived macrophages were generated from MYD88 knock-out mice and wild type littermate controls. Macrophages were stimulated with either 1 µg/ml Pam_3_CSK_4_, 100 ng/ml LPS, 1 µg/ml FSL1 or 2.5 µM ODN1826 for 1 h. Cell lysates were prepared and subjected to immunoblot with the indicated antibodies. (**B**) As in **A** except that macrophages were generated from TRIF knock-out mice and wild type littermate controls. All immunoblots are representative of at least two independent experiments.

To verify that the MyD88 pathway was critical for enabling LRRK2 to become phosphorylated at Ser935, we generated BMDMs from littermate wild type and MyD88 knock-out mice and observed that the TLR agonists Pam_3_CSK_4_ (TLR1/TLR2), FSL1 (TLR6) and ODN1826 (TLR9) failed to stimulate phosphorylation of Ser935 in MyD88 knock-outs (Fig 4A). Although LPS activated the TRIF pathway in MyD88-deficient BMDM macrophages, emphasised by phosphorylation of p38 and JNK, LPS failed to induce Ser935 phosphorylation (Fig 4A). We also observed that the basal phosphorylation of Ser935 was similar in non-stimulated wild type and MyD88 knock-out macrophages indicating that a MyD88-independent pathway controls basal phosphorylation (Fig 4A). We also found in TRIF knock-out BMDMs that Ser935 phosphorylation is increased to the same extent as in wild-type macrophages following LPS stimulation, further suggesting that the TRIF pathway is not regulating Ser935 phosphorylation (Fig 4B).

### LRRK2 Kinase Activity is not Required for TLR Mediated Ser935 Phosphorylation

**Figure 5:**
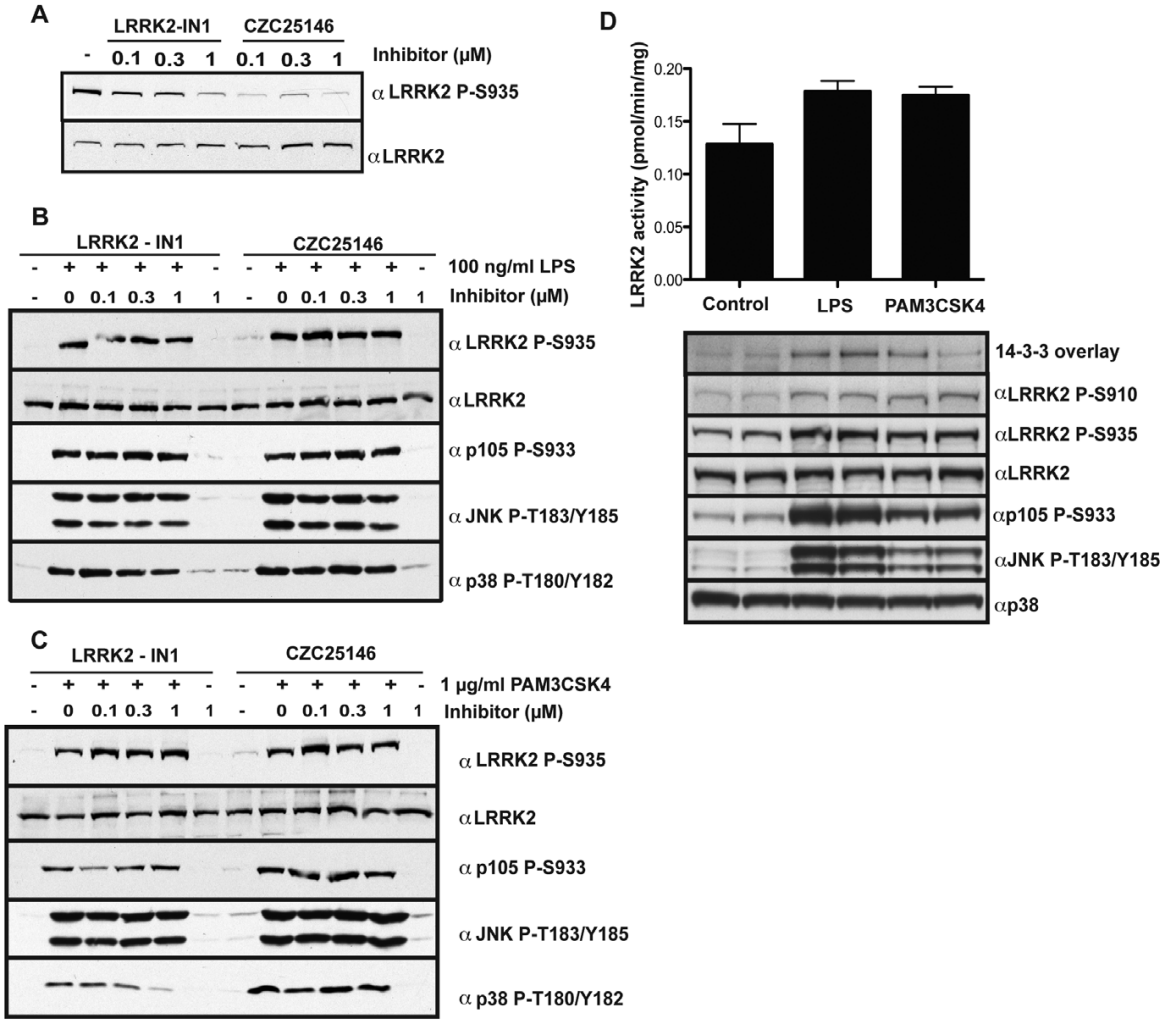
Ser935 phosphorylation during TLR signaling does not require LRRK2 kinase activity. (**A**) Primary bone marrow derived macrophages were treated with indicated concentrations of LRRK2-IN1 or CZC25146 or DMSO as control for 1 h. Cell lysates were prepared and subjected to immunoblot with the indicated antibodies. (**B**) Primary bone marrow derived macrophages were pre-treated with indicated concentrations of LRRK2-IN1 or CZC25146 or DMSO as control for 1 h before stimulation with 100 ng/ml LPS for 1h. Cell lysates were prepared and subjected to immunoblot with the indicated antibodies. (**C**) As in **B** except 1 µg/ml Pam_3_CSK_4_ was used. (**D**) RAW264.7 cells were treated with 100 ng/ml LPS or 1 µg/ml Pam_3_CSK_4_ for 1 h before cell lysis. Endogenous LRRK2 was immunoprecipitated from 3 mg RAW264.7 cell lysate with 3 µg rabbit monoclonal LRRK2 100–500 antibody. Kinase activity was measured using 20 µM Nictide with n = 4 in duplicate. Kinase activity is reported as pmol of ATP incorporated into Nictide per minute per mg of lystate immunoprecipitated from. A parallel set of immunoprecipitations was used to measure 14-3-3 binding by overlay assay. All results are representative of at least two independent experiments.

As mentioned in the introduction treatment of cells or mice with LRRK2 kinase inhibitors induces dephosphorylation of LRRK2 at Ser910 and Ser935 [10], [11]. We therefore explored the effect that the LRRK2 kinase inhibitors, LRRK2-IN1 and CZC25146, had on basal as well as MyD88 pathway-induced Ser935 phosphorylation observed in BMDMs. Consistent with previous observations, treatment of non-stimulated BMDMs with LRRK kinase inhibitors, reduced the basal level of LRRK2 Ser935 phosphorylation to barely detectable levels (Fig 5A). In contrast however, LRRK2-IN1 had no effect on Ser935 phosphorylation induced by LPS or Pam_3_CSK_4_ (Fig 5B). Likewise CZC25146 failed to block the LPS or Pam_3_CSK_4_ phosphorylation of LRRK2 Ser935 in BMDMs at a number of concentrations tested (Fig 5C). We also investigated the effect of LRRK2-IN1 in RAW264.7 cells. Consistent with the results from BMDMs, LRRK2-IN1 reduced the basal phosphorylation of Ser935, as well as Ser910, but LRRK2-IN1 failed to suppress LPS-induced phosphorylation of these residues (Fig S2). This indicates that, unlike basal Ser910 and Ser935 phosphorylation, the phosphorylation induced by LPS and Pam_3_CSK_4_ is not regulated by LRRK2 kinase activity.

To examine the direct effect of LPS and Pam_3_CSK_4_ on LRRK2 kinase activity we employed the murine macrophage cell line RAW264.7, as we were unable to culture sufficient BMDMs to assess LRRK2 catalytic activity robustly. Increased phosphorylation of Ser935 following treatment of RAW264.7 cells with LPS or Pam_3_CSK_4_ resulted in increased binding of 14-3-3 in a far Western assay, however, the kinase activity of immunoprecipitated LRRK2 did not differ significantly (Fig 5D). This is consistent with our previous work that indicated that phosphorylation of Ser910/Ser935 and 14-3-3 binding had no impact on LRRK2 protein kinase activity [8].

### TBK1 and IKKε Contribute to MYD88-pathway Mediated Phosphorylation of LRRK2

**Figure 6:**
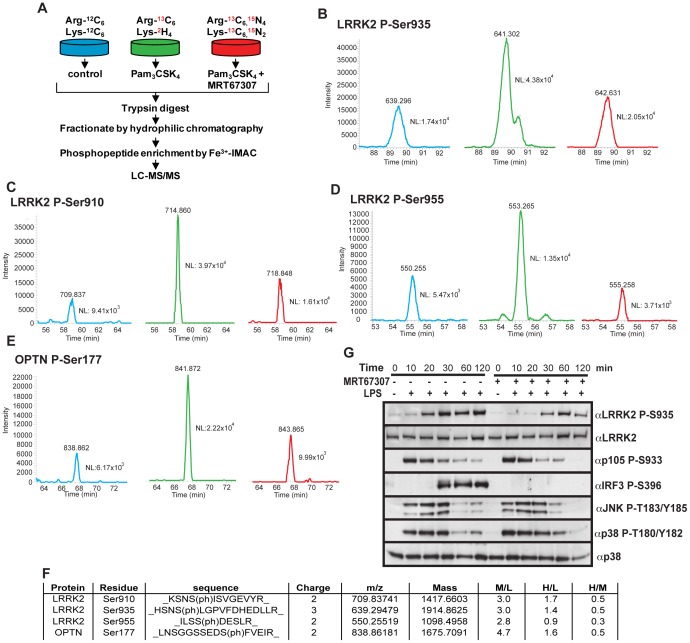
TBK1 and IKKε regulate LRRK2 Ser935 phosphorylation following TLR activation. A ) Schematic of SILAC experiment. **B-E**) Extracted Ion Current for phosphopeptides encompassing Ser935 (**B**), Ser910 (**C**) and Ser955 (**D**) of LRRK2 and Ser177 of optineurin (**E**). The results for unstimulated RAW264.7 macrophages are presented in blue, results for RAW264.7 macrophages stimulated for 60 min with Pam_3_CSK_4_ (1 µg/ml) are in green and the results from RAW264.7 macrophages pre-treated with 2 µM MRT67307 prior to stimulation with Pam_3_CSK_4_ (1 µg/ml) for 60 min are depicted in red. (**F**) Table summarizing the results of the phosphopeptides from LRRK2 identified in the phosphoproteomics screen. **G**) Primary bone marrow derived macrophages were pre-treated with 2 µM MRT67307 or DMSO control for 1 h before stimulation with 100 ng/ml LPS for the indicated time points. Immunoblots are representative of at least two independent experiments.

We next investigated how inhibition of TBK1 and IKKε impacted on LRRK2 phosphorylation, using an inhibitor termed MRT67307 [22]. We employed a quantitative stable isotope labelling with amino acids in cell culture (SILAC) mass spectroscopy approach to study LRRK2 phosphorylation in RAW264.7 macrophages that were stimulated in the presence or absence of Pam_3_CSK_4_ and MRT67307 (Fig. 6A). After enrichment of the phosphopeptides using sequential hydrophilic interaction chromatography and immobilized metal ion affinity chromatography, we were able to identify three LRRK2 derived phosphopeptides encompassing Ser910, Ser935 and Ser955. In addition we also identified a peptide encompassing Ser177 of optineurin a known TBK1/IKKε substrate [35] (Fig 6B, 6C, 6D, 6E, 6F). Pam_3_CSK_4_ significantly enhanced phosphorylation of Ser935, which was partially inhibited by MRT67307 (Fig 6B). Interestingly, Pam_3_CSK_4_ also induced phosphorylation of Ser910 and Ser955 as well as optineurin at Ser177, and the phosphorylation of all these residues was similarly inhibited by MRT67307 (Fig 6C, 6D, 6E, 6F). This suggests that TBK1/IKKε may also regulate the phosphorylation of Ser910 and Ser955 in addition to Ser935.

Employing a phosphospecific Ser935 antibody we also confirmed in BMDMs that following LPS stimulation, under conditions in which MRT67307 ablated phosphorylation of the TBK1/IKKε substrate IRF3 at Ser396, it also partially reduced the phosphorylation of LRRK2 at Ser935 (Fig 6G). The inhibitory effect of MRT67307 was most pronounced at the 20–30 min time points of LPS treatment (Fig 6G).

### IKKα and IKKβ Contribute to MyD88-pathway Mediated Phosphorylation of LRRK2

**Figure 7:**
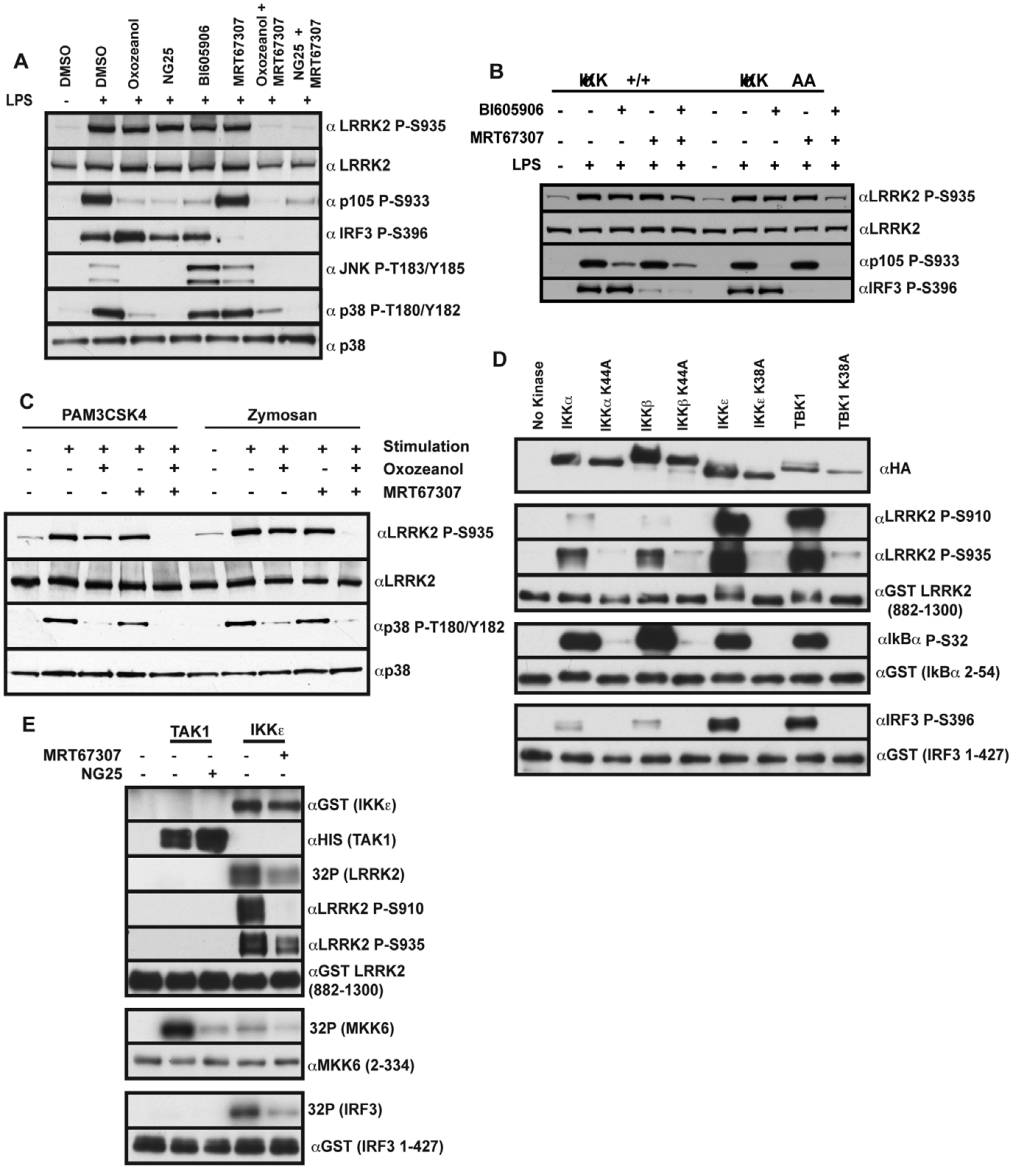
IKKα and IKKβ contribute to LRRK2 Ser935 phosphorylation following TLR activation. (**A**) Primary bone marrow derived macrophages were treated with either DMSO, 1 µM 5z-7-Oxozeanol (TAK1 inhibitor), 2 µM NG25 (TAK1 inhibitor), 2 µM MRT67307 (TBK1/IKKε inhibitor) or 10 µM B1605906 (IKKβ inhibitor) alone or in the indicated combinations for 1 h before stimulation with 100 ng/ml LPS for 1 h. Cell lysates were subjected to immunoblot analysis with the indicated antibodies. (**B**) Bone marrow derived macrophages were generated from kinase inactive IKKα double Ser176A and Ser180A knock-in mice or littermate wild type controls. Macrophages were treated with either DMSO, 2 µM MRT67307 (TBK1/IKKε inhibitor) or 10 µM B1605906 (IKKβ inhibitor) as indicated for 1 h before stimulation with 100 ng/ml LPS for 1 h. Cell lysates were subjected to immunoblot analysis with the indicated antibodies. (**C**) As in **A** except that BMDMs were treated with DMSO, 1 µM 5z-7-Oxozeanol (TAK1 inhibitor), 2 µM MRT67307 (TBK1/IKKε inhibitor) alone or in combination for 1 h before stimulation with 1 µg/ml Pam_3_CSK4 or 200 µg/ml zymosan. (**D**) Ha tagged wild type and kinase inactive variants of IKKα IKKβ IKKε and TBK1 were expressed by transfection into HEK293 cells then immunoprecipitated by Ha-agarose. Kinases were used to phosphorylate bacterially expressed substrates GST-LRRK2 (882-1300), GST-IκBα (2-54) and GST-IRF3 (1-427) at 30°C for 30 min before reaction termination with sample buffer. 10% of the in vitro phosphorylation reaction was used for immunoblot analysis with the indicated antibodies. **E**) Baculovirus expressed TAK1 and IKKε were used to phosphorylate bacterially expressed substrates GST-LRRK2 (882-1300), GST-MKK6 (2-334) and GST-IRF3 (1-427) at 30°C for 30 min before reaction termination with sample buffer. 10% of the in vitro phosphorylation reaction was used for immunoblot analysis with the indicated antibodies or for autoradiograph.

The finding that MRT67307 only partially inhibited LPS or Pam_3_CSK_4_ induced phosphorylation of Ser935 at earlier time points, suggested that other MyD88-activated protein kinases were involved in phosphorylating Ser935. To investigate whether the related IKKα and IKKβ could contribute to phosphorylation of Ser935, we treated cells with MRT67307 (to inhibit TBK1/IKKε) in combination with either of two structurally unrelated kinase inhibitors that inhibit the TAK1 protein kinase termed NG25 or 5Z-7-oxozeaenol and therefore also inhibit activation of IKKα and IKKβ. The structure of NG25 and its method of synthesis is shown in method S1. NG25 inhibited TAK1 in vitro with an IC50 of 4 nM and has previously been shown to inhibit TAK1 in cells [36]. The selectivity of NG25 against numerous kinases was measured by ActivX KiNativ profiling [37] (Table S1), Ambit profiling (Table S2) or radioactive filter binding assay performed at the International Centre for Kinase Profiling (Table S3) showing that NG25 is a relatively specific inhibitor for TAK1. When either 5Z-7-oxozeaenol or NG25 were used in combination with MRT67307, LPS induced phosphorylation was completely prevented (Fig 7A). In contrast, treatment of BMDMs with either NG25 or 5Z-7-oxozeaenol in the absence of MRT67307 had no significant effect on Ser935 phosphorylation following stimulation with LPS, under conditions where these compounds inhibited p105, JNK and p38 activation (Fig 7A).

We also used an IKKβ inhibitor, BI605906 [22], in combination with MRT67307 and found that this did not reduce LPS mediated phosphorylation of Ser935 any more than MRT67307 employed alone (Fig 7B), indicating that IKKα might be responsible for residual phosphorylation. To test this we treated BMDMs derived from knock-in mice expressing catalytically inactive IKKα with MRT67307 (to inhibit IKKε and TBK1) and BI605906 (to inhibit IKKβ). This ablated phosphorylation of Ser935 (Fig 7B), supporting the view that all four IKK’s contribute to LRRK2 Ser935 phosphorylation in macrophages.

As mentioned above Zymosan also induces phosphorylation of LRRK2 at Ser935, probably vial TLR2 but not the dectin-1 pathway (Fig 3D). Consistent with this treatment of BMDMs with both the TAK1 5Z-7-oxozeaenol inhibitor and the TBK1/IKKε inhibitor MRT67307 was required to block phosphorylation of LRRK2 at Ser935 induced by Zymosan (Fig 7C).

### IKKα, IKKβ, TBK1 and IKKε Phosphorylate LRRK2 at Ser910 and Ser935 in vitro

We next expressed wild type and kinase-inactive mutants of HA epitope tagged IKKα, IKKβ, TBK1 and IKKε in HEK293 cells and tested whether these could phosphorylate Ser935 as well as Ser910 employing phosphospecific antibodies that recognise these residues. This revealed that all four wild type, but not similar levels of kinase-inactive, IKK isoforms phosphorylated LRRK2 at Ser910 and Ser935 in vitro. TBK1 and IKKε phosphorylated LRRK2 to a markedly greater extent than IKKα and IKKβ under conditions in which all four IKK isoforms phosphorylated IκBα at Ser32 to a similar extent (Fig 7D). We also tested whether baculovirus expressed TAK1 was able to phosphorylate LRRK2 and found that although TAK1 phosphorylated a known substrate MKK6, it failed to phosphorylate LRRK2 at Ser910 or Ser935 (Fig 7E).

### IKK-mediated Cytokine Signaling is not Impaired in LRRK2 Deficient Macrophages

**Figure 8:**
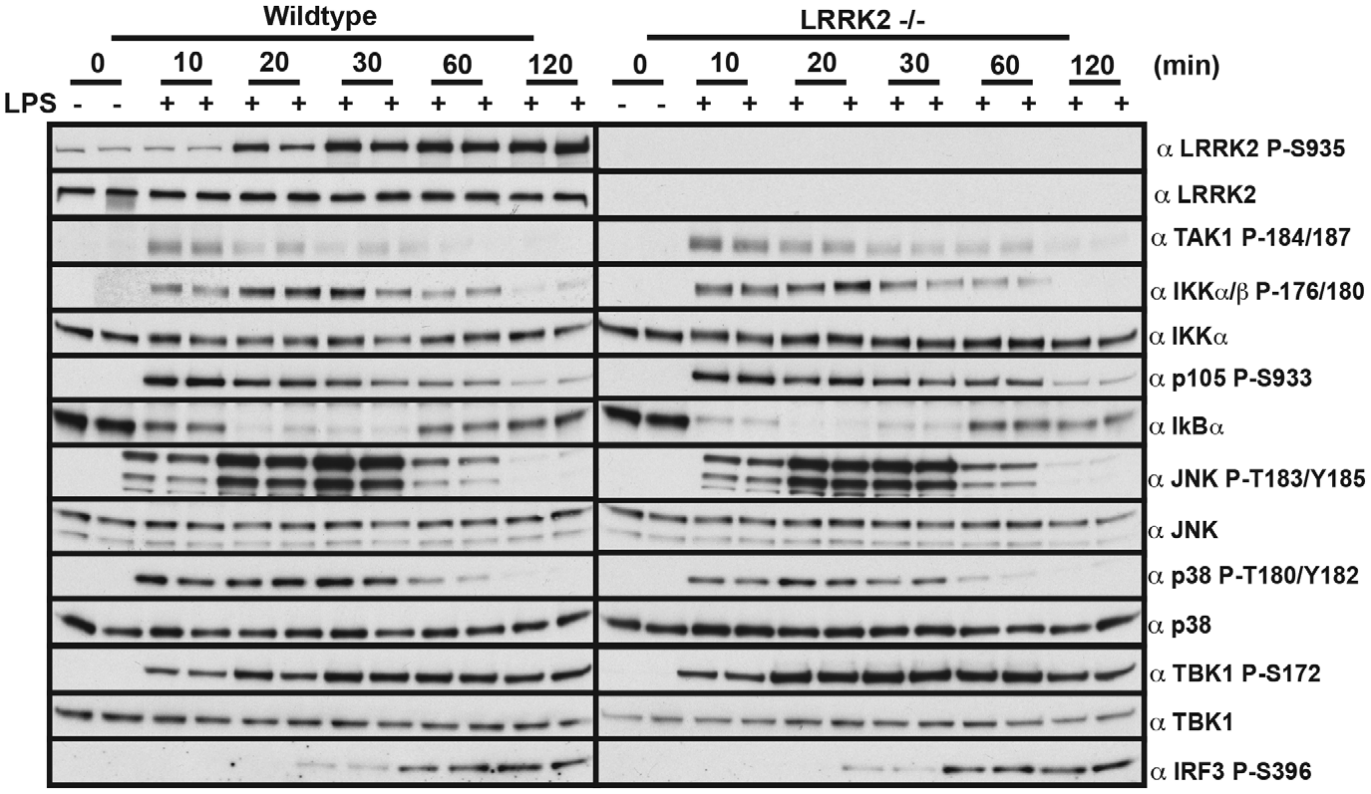
Acute IKK signalling is not impaired in LRRK2 knock-out macrophages. Bone marrow derived macrophages were prepared from LRRK2 knock-out mice and littermate controls. BMDMs were treated with 100 ng/ml LPS for the indicated time points. Cells lysates were subjected to immunoblot with the indicated antibodies. Results are representative of two independent experiments performed in duplicate.

We next examined cytokine secretion (IL-6, keratinocyte chemoattractant, RANTES, IL-1β, Monocyte chemoattractant protein 1, IL-10, TNFα and IL-12 (p40)) in wild type or LRRK2 knock-out BMDMs at 0 h, 4 h, 8 h and 12 h following stimulation with LPS (Table 1) or Pam_3_CSK_4_ (Table 2). We also measured mRNA expression of cytokines at 0 h, 4 h, 8 h and 12 h post stimulation with LPS (Table 3) or Pam_3_CSK_4_ (Table 4). As expected LPS and Pam_3_CSK4 induced profound increases in cytokine secretion and cytokine mRNA expression, but no consistent significant differences were observed between the wild type and LRRK2 knock-out BMDMs (Tables 1, 2, 3, 4). Additionally we found that there were no differences in the time course of LPS-induced phosphorylation of TAK1, IKKα/IKKβ, p105, IκB, JNK, p38, TBK1 or IRF3 in wild type and LRRK2 knock-out BMDMs (Fig 8).

**Table 1:** Cytokine secretion in wild type and LRRK2 KO BMDM’s following LPS stimulation.

Analyte	Time = 0		Time = 4 h		Time = 8 h		Time = 12 h	
	WT	KO	WT	KO	WT	KO	WT	KO
IL-6	ND	ND	209±86	231±26	1531±110	1278±199	2107±127	1592±210
KC	ND	ND	944±64	690±165	1045±54	725±136	1245±162	901±114
RANTES	5.95±1.28	4.06±1.00	617±75	587±78	5203±266	4887±734	14043±1653	11362±1206
IL-1β	ND	ND	ND	ND	9.90±2.27	9.36±2.16	12.74±2.31	16.82±6.74
MCP1	ND	ND	4524±253	4841±589	9336±461	7377±740	10998±1875	14937±2006
IL-10	5.41±0.34	4.28±0.79	99.2±10.3	135.9±30.2	479±26	403±76	346.8±14.4	448.5±84.3
TNFα	ND	ND	6220±562	8641±998	3934±233	2851±333	2122±47	1831±163
IL-12 (p40)	ND	ND	189±16	186±43	1455±109	1192±154	2688±316	2347±308

Bone marrow derived macrophages from LRRK2 knockout mice and wild type littermate controls were plated at a density of 1×10^6^ in 2 ml of tissue culture media. Cells were treated with 100 ng/ml LPS 24 h after plating and media collected at the indicated time points. Cytokines were measured with an antibody based multiplex assay and concentrations determined by standard curve. Data is presented as mean concentration in pg/ml ± S.E.M. Experiments were performed with n = 4 in duplicate.

**Table 2:** Cytokine secretion in wild type and LRRK2 KO BMDM’s following PAM3CSK4 stimulation.

Analyte	Time = 0		Time = 4 h		Time = 8 h		Time = 12 h	
	WT	KO	WT	KO	WT	KO	WT	KO
IL-6	ND	ND	18.6±6.1	19.1±7.8	1965±337	1701±363	6470±1755	8470±1688
KC	ND	ND	947±132	528±133	8033±1426	5350±1139	17238±3799	13608±5972
RANTES	5.95±1.28	4.06±1.00	45.3±10.9	35.6±11.2	836±213	707±123	3057±864	2402±267
IL-1β	ND	ND	ND	ND	7.58±1.92	9.36±2.16	25.0±5.69	21.5±5.92
MCP1	ND	ND	164±78	95.8±25	329±99	245±83	1945±276	971±282
IL-10	5.41±0.34	4.28±0.79	63.2±7.3	51.9±12.0	158±16	140±25	172±10	135±27
TNFα	ND	ND	995±207	839±56	3007±271	2223±395	2454±170	2281±181
IL-12 (p40)	ND	ND	17.2±0.57	16.1±2.09	188±33	168±27	663±66	524±42

Bone marrow derived macrophages from LRRK2 knockout mice and wild type littermate controls were plated at a density of 1×10^6^ in 2 ml of tissue culture media. Cells were treated with 1 µg/ml Pam_3_CSK_4_ 24 h after plating and media collected at the indicated time points. Cytokines were measured with an antibody based multiplex assay and concentrations determined by standard curve. Data is presented as mean concentration in pg/ml ± S.E.M. Experiments were performed with n = 4 in duplicate.

**Table 3:** mRNA expression in wild type and LRRK2 KO BMDM’s following LPS stimulation.

Gene	Time = 0		Time = 4h		Time = 8h		Time = 12h	
	WT	KO	WT	KO	WT	KO	WT	KO
IL-6	1.0±0.12	1.1±0.09	8026±1068	10128±2939	74763±7988	70365±6522	69450±8032	76367±6240
IL-1β	1.0±0.17	1.0±0.11	1063±106	780±174	1710±173	1750±129	786±64	843±97
TNFα	1.0±0.04	1.3±0.08	53±2.2	63±5.1	33±2.5	30±5.3	16±1.2	13±0.84
IFNβ	1.0±0.24	1.4±0.35	8295±2299	10050±1291	2232±297	2803±335	649±165	932±82
IL-12(p70)	1.0±0.11	1.1±0.71	8627±958	7122±727	2922±228	2951±240	1425±58	1942±144
IL-12 (p40)	1.0±0.57	1.1±0.29	24361±3392	25180±1050	17610±1518	12671±2077	7206±645	9804±660

Bone marrow derived macrophages from LRRK2 knockout mice and wild type littermate controls were plated at a density of 1×10^6^ in 2 ml of tissue culture media. Cells were treated with 100 ng/ml LPS 24 h after plating and RNA collected at the indicated time points. Gene expression was normalized to GAPDH and the fold increase in mRNA expression compared to wild type macrophages at time 0 was calculated by the comparative Ct method (2^−ddct^). Data is presented as mean fold increase in expression ± S.E.M. Experiments were performed with n = 4 in duplicate.

**Table 4:** mRNA expression in wild type and LRRK2 KO BMDM’s following PAM3CSK4 stimulation.

Gene	Time = 0		Time = 4 h		Time = 8 h		Time = 12 h	
	WT	KO	WT	KO	WT	KO	WT	KO
IL-6	1.0±0.24	1.1±0.09	3247±838	3377±548	22791±5384	20405±4896	20866±5205	17094±4900
IL-1β	1.0±0.28	1.0±0.13	766±182	752±113	2643±336	2491±139	1928±292	1543±255
TNFα	1.0±0.30	1.0±0.04	19.3±3.8	18.7±1.0	7.8±1.8	7.2±0.23	2.97±0.69	2.58±0.76
IFNβ	1.0±0.35	1.3±0.43	15.5±3.7	19.0±4.3	26.8±6.9	25.9±4.3	9.9±2.9	9.6±1.5
IL-12(p70)	1.0±0.40	1.1±0.34	96±25	94±10	205±47	196±7	211±55	168±49
IL-12(p40)	1.0±0.30	1.1±0.43	1680±428	1558±131	3050±876	2585±215	2920±814	2070±428

Bone marrow derived macrophages from LRRK2 knockout mice and wild type littermate controls were plated at a density of 1×10^6^ in 2 ml of tissue culture media. Cells were treated with 1 µg/ml Pam_3_CSK_4_ 24 h after plating and RNA collected at the indicated time points. Gene expression was normalized to GAPDH and the fold increase in mRNA expression compared to wild type macrophages at time 0 was calculated by the comparative Ct method (2^-ddct^). Data is presented as mean fold increase in expression ± S.E.M. Experiments were performed with n = 4 in duplicate.

## Discussion

In light of recent findings that LRRK2 is highly expressed in immune cells and that the expression of LRRK2 can be further modulated by IFNγ [13], we utilised novel and highly sensitive LRRK2 antibodies to investigate the expression and phosphorylation of LRRK2 in response to agonists of innate immune signalling pathways. The key finding of this study is that all MyD88 agonists tested trigger marked phosphorylation of LRRK2 at the 14-3-3 binding sites Ser910 and Ser935. Monitoring phosphorylation of LRRK2 in RAW264.7 macrophages (Fig 6), we find evidence that phosphorylation of Ser955 is enhanced by Pam3CSK4 and inhibited with the TBK1/IKKε inhibitor MRT67307 similarly to Ser910 and Ser935. A recent report suggests treatment of cells with LRRK2 induces dephosphorylation of Ser910, and Ser935 as well as Ser955 and Ser973 [12], [38] and the amino acid sequence surrounding these residues are similar. Taken together with the data presented in this paper this suggests that these residues are similarly regulated. Further work will be required to determine whether the phosphorylation of S955 and S973 in vivo is regulated by IKKs when state of the art phospho-specific antibodies for these residues become readily available.

Our data suggest that phosphorylation of Ser910 and Ser935 is likely to be mediated directly by the canonical as well as non-canonical IKK kinases. Not only do these enzymes directly phosphorylate Ser935 as well as Ser910 in vitro, but combinations of inhibitors that prevent activation of these enzymes also inhibited LPS-stimulated phosphorylation of these sites in vivo. In vitro, LRRK2 was phosphorylated to a greater extent by the IKK related kinases (TBK1 and IKKε) rather than the canonical IKKs (IKKα and IKKβ). Also inhibition of TBK1 and IKKε using MRT67307 resulted in a partial inhibition of Ser935 phosphorylation whilst inhibition of the activation of IKKα and IKKβ with TAK1 inhibitors had no effect alone on LRRK2 phosphorylation. The relative contribution of each IKK to phosphorylation of Ser910, Ser935 and potentially S955 remains to be determined. Our data does not rule out the possibility that other kinases that we have not identified and that might be regulated by IKKs, control phosphorylation of Ser910, Ser935 and Ser955. PKA has been suggested as a kinase capable of phosphorylating LRRK2 at Ser935 in vitro and by transfection in cell culture [9], [39] however, validation of PKA as an upstream kinase for LRRK2 remains to be demonstrated at the endogenous level.

Recent work has uncovered other substrates that can be phosphorylated by the four IKK isoforms. These include NEMO and TANK [22] where inhibition of both the TBK1/IKKε and IKKα/IKKβ branches of the MyD88 pathway are required to suppress phosphorylation. Optineurin is also phosphorylated by the 4 IKK members, but in this case the canonical and non-canonical IKKs phosphorylate different residues (IKKα/IKKβ-Ser513 and TBK1/IKKε-Ser177) preferentially [35]. In the case of optineurin, Ser177 was phosphorylated when macrophages were stimulated with Poly(I:C) a TLR3 agonist that signals via TRIF, or the TLR1/2 agonist Pam_3_CSK4 [35] which signals via MyD88. This differs from Ser935 of LRRK2, which is only phosphorylated when TBK1/IKKε are activated by the MyD88 signalling pathway. In contrast, IRF3, is only phosphorylated at Ser396 when TBK1/IKKε are activated via the TRIF-dependent pathway [23], [24]. These observations provide further evidence that the substrates that TBK1/IKKε phosphorylate are determined by the pathway that leads to their activation. This could be explained if the MyD88 and TRIF pathways activate distinct pools of TBK1/IKKε, which are targeted to specific subsets of substrates. To our knowledge LRRK2 is the first substrate to be reported that is phosphorylated by all four IKK family members in only a MyD88-dependent manner.

That LRRK2 is phosphorylated during inflammatory signalling is of interest as increasing evidence suggests that inflammation is linked to the progression of neurodegeneration and Parkinson’s disease [38], [40], [41]. Increased IL-6 and increased TNFα have been found in the brains, CSF and serum of patients with PD [42]–[46]. In PD, increased inflammatory cytokine secretion is associated with the activation of microglia, the resident macrophages of the brain [47]. In rodent models, activation of microglia with either central or peripheral LPS induces loss of dopaminergic substantia nigra neurons [48]–[50]. Neuroinflammation is also observed in other routinely used PD animal models including the 6-hydroxy-dopamine and MPTP models [51], [52]. In the 6-hydroxydopamine model, inhibiting tumour necrosis factor signaling can attenuate loss of dopaminergic neurons [53].

Intriguing recent studies also demonstrate that mutations in LRRK2 are significant risk factors in Crohn’s disease, an inflammatory disease of the bowel [54]–[56]. Supporting a role of LRRK2 in Crohn’s disease, LRRK2 knock-out macrophages display increased production of the inflammatory cytokines IL-12 and IL-6 when stimulated with the yeast cell wall glucan component, zymosan, rendering these mice more susceptible to inflammatory bowel disease in a mouse model [16]. Although zymosan activates both TLR2 and dectin-1, evidence suggests that regulation of NFAT by zymosan is mediated via dectin-1 [57]. Curdlan, a specific activator of dectin-1, that does not activate TLR2, did not increase the phosphorylation of LRRK2 at Ser935 (Fig 3F). Consistent with the effects of zymosan-stimulated phosphorylation of LRRK2 being mediated via a TLR2-MYD88 pathway, we found that the ability of zymosan to induce phosphorylation of Ser935 was prevented by inhibition of both TBK1/IKKε with MRT67307 and TAK1 with 5Z-7-oxozeaenol (Fig 7C). If the effects of zymosan on NFAT localisation are mediated through the dectin-1 pathway, this would indicate that LRRK2 phosphorylation at serines 910 and 935 by the MyD88 pathway is not involved in controlling NFAT. The finding that LRRK2 knock-out had no effect on either Pam_3_CSK4 or LPS-induced inflammatory cytokine expression and secretion also indicates that NFAT is unlikely to be controlled directly by LRRK2 in the TLR signalling network.

More work is required to uncover how phosphorylation of LRRK2 by the IKK family kinases regulates function and impacts on macrophage biology. It may be necessary to generate LRRK2 knock-in mice that cannot be phosphorylated by TBK1/IKK isoforms to address this question. A number of recent studies have implicated both TBK1 [58], [59] and LRRK2 in controlling autophagy [60]–[62]. We have undertaken some initial studies in wild type and LRRK2 knock-out BMDMs stimulated with LPS or starved of amino acids looking at various autophagic markers, such as levels of LC3 lipidation and p62 levels. However, thus far we have not observed any significant differences between wild type and LRRK2 knock-out macrophages (FIV and Ian Ganley unpublished).

A final aspect of this work relates to the use of phospho-specific antibodies recognising phosphorylated Ser910 and Ser935 as biomarkers for LRRK2 inhibitors. As dephosphorylation of LRRK2 at Ser935 and Ser910 is being used to evaluate the efficacy of LRRK2 inhibitors, the work described in this study reveals some limitations to our previous hypothesis relating to using dephosphorylation of these residues as pharmacodynamic biomarkers [10]. We confirmed in BMDMs, observations we have made in many other cell types, namely that the basal phosphorylation of Ser910 and Ser935 is markedly reduced following incubation with LRRK2 inhibitors. The finding that phosphorylation of Ser910 and Ser935 induced by the MyD88 pathway is not sensitive to LRRK2 inhibitors indicates that caution is needed when using this assay to assess the efficacy of LRRK2 inhibitors in cells that express TLR receptors, under conditions in which this pathway may be activated. For example, if phosphorylation of Ser910 and Ser935 is being used as a readout to assess efficacy of LRRK2 inhibitors in vivo it is essential that conditions are used in which the MyD88 pathway is not activated. It should be noted that inhibitors of IKKs could not reduce the constitutive basal phosphorylation of LRRK2.

In conclusion, we provide the first direct evidence that links the IKKs with the LRRK2 kinase. Our results provide evidence that the canonical as well as the non-canonical IKKs can phosphorylate Ser935 and Ser910 and probably also Ser955, but only after activation by the MyD88 pathway. This provides further evidence that LRRK2 plays a role in macrophages, which will be important to define in future, work. It will also be exciting to determine whether defects in LRRK2 signalling in immune cells are linked to the development of Parkinson’s disease in patients with LRRK2 mutations.

## Supporting Information

Figure S1 **TLR agonists increase LRRK2 phosphorylation at Serines 910 and 935 in RAW 264.7 cells.** RAW 264.7 macrophages were treated with the following TLR agonists for 1 h. 1 µg/ml Pam3CSK4, 10^8^ cells HKLM, 10 µg/ml LMW and HMW Poly(I:C), 100 ng/ml LPS, 10 µg/ml Flagellin, 1 µg/ml FSL1, 1 µM CLO97 and 2.5 µM ODN1826. Lysates were subjected to immunoblot with the indicated antibodies. Results are representative of at least two independent experiments.
(TIF)

Figure S2 **Effect of LRRK2-IN1 on LRRK2 phosphorylation in RAW cells.** RAW 264.7 macrophages were pre-treated with 1 µM LRRK2-IN1 or DMSO as control for 1 h then stimulated plus or minus 100 ng/ml LPS for 1 h. Cell lysates were prepared and subjected to immunoblot with the indicated antibodies. Results are representative of at least two independent experiments.
(TIF)

Method S1 **Synthesis of NG25.**
(DOC)

Table S1 **ActivX selectivity profiling of NG25.** Cellular kinase selectivity as assessed using the KiNativ(tm) technology [37]. Percent inhibition (color coded as indicated in the legend) of kinase labeling by ATP-biotin that results from incubating HUH7 cells with the inhibitors at concentrations of 1 µM and 10 µM is indicated (larger numbers indicate stronger binding to the kinase).
(XLSX)

Table S2 **Ambit selectivity profiling of NG25.** Inhibitors were screened at a single concentration of 10 µM. Scores are related to the probability of a hit and are not strictly an affinity measurement. At a screening concentration of 10 µM, a score of less than 10% implies that the false positive probability is less than 20% and that the Kd value is most likely less than 1 µM. A score between 1 and 10% implies that the false positive probability is less than 10%, although it is difficult to assign a quantitative affinity from a single-point primary screen. A score of less than 1% implies that the false positive probability is less than 5% and that the Kd value is most likely less than 1 µM.
(XLSX)

Table S3 **International centre for kinase profiling of NG25.** The specificity of NG25 was examined against a panel of over 100 kinases by the International Centre for Protein Kinase Profiling, MRC Protein Phosphorylation Unit, University of Dundee (www.kinase-screen.mrc.ac.uk) at concentrations of 0.1, 1 and 10 µM. The profiling of NG25 at 0.1 µM has been published previously [36] however, we include it again for ease of comparison with the other screening data. The results for each protein kinase tested are expressed as percentage activity remaining following inhibitor treatment compared to no inhibitor controls.
(XLSX)

Table S4 **qRT-PCR primers.** Sense and anti-sense primer sequences for the analysis of inflammatory gene expression by qRT-PCR are listed.
(XLSX)
