# Expression of Concern: The IkappaB Kinase Family Phosphorylates the Parkinson’s Disease Kinase LRRK2 at Ser935 and Ser910 during Toll-Like Receptor Signaling

**DOI:** 10.1371/journal.pone.0330958

**Published:** 2025-08-26

**Authors:** The PLOS One Editors

After publication of this article [1], concerns were raised regarding results presented in Figs 4 and 8.

Specifically:

- The Fig 4A αLRRK2 panel appears similar to the Fig 4B αLRRK2 panel despite representing different experimental results.
- There appears to be a vertical discontinuity in Fig 8 Wildtype α IKKα/β P-176/180 between lanes 1 and 2.

PLOS was contacted by the Research Integrity Group from the School of Life Sciences at the University of Dundee regarding the concerns raised about the αLRRK2 panels in Fig 4A and 4B. They reviewed the relevant laboratory notebooks and primary data leading them to conclude that the Fig 4A αLRRK2 panel is incorrect and that the Fig 4B αLRRK2 panel is correct. They provided photographs of the original films underlying the Fig 4A and 4B αLRRK2 panels (S1–S2 Files). However, they noted that in the replacement Fig 4A αLRRK2 data (S1 File), the signal for LRRK2 in lane 1 corresponding to the Wild type CON sample was very low, and they suggested that this may have been caused by incomplete protein transfer at the edge of the gel/blot. They stated that the same Wildtype CON sample was also used to produce Fig 4B and that in Fig 4B, LRRK2 expression is evident in the Wild type CON sample. PLOS remains concerned for the reliability of the replacement Fig 4A αLRRK2 blot in S1 File due to the reduction of visible LRRK2 protein expression in the Wildtype CON lane.

The legend of Fig 4 in [1] states “All immunoblots are representative of at least two independent experiments”; however, the Research Integrity Group from the School of Life Sciences at the University of Dundee located only one set of blots in the available underlying data (S1–S5 Files).

For the Fig 4B αIRF3 P-S396 and αp38 panels, the corresponding authors stated that the x-ray films corresponding to the published blots are no longer available. They provided separate western blots in support of the Fig 4B αIRF3 P-S396 panel (S4 File) and the Fig 4B αp38 panel (S5 File). The corresponding author ND stated that these blots were conducted at the same time and with the same samples as the rest of the immunoblots in Fig 4. The *PLOS One* Editors noted that the lane order of the western blot in S4 File appears different to the published Fig 4B αIRF3 P-S396 blot, with fewer sample lanes shown in S4 File, and what appears to be a protein ladder in the position of the Wild type CON lane in the Fig 4B αIRF3 P-S396 panel in [1]. The corresponding authors stated that in all experiments in Fig 4, 10-lane gels were used, and that protein ladder was loaded into the first well along with the first protein sample (Wild type CON). They stated that in S4 File, the 50 kDa ladder partially overlaps with the protein region of interest, and both ladder and WT CON are present in the same lane. Given the ladder cannot be differentiated from the protein of interest in S4 File, PLOS remain concerned about the reliability of the Wild type CON results in Fig 4.

Regarding the vertical discontinuity in the Fig 8 Wildtype α IKKα/β P-176/180 western blot, the corresponding author provided a photograph of the original Wildtype α IKKα/β P-176/180 film (S6 File) which shows that the apparent vertical discontinuity is due to changes in background. PLOS therefore considers this concern resolved.

The remaining underlying data were not provided for editorial review. The Research Integrity Group from the School of Life Sciences at University of Dundee stated that the underlying blot data are available from them upon request.

The *PLOS One* Editors issue this Expression of Concern to notify readers of the above issues, and to relay the available information and data provided.

## Supporting information

S1 File Replacement data for Fig 4A αLRRK2 panel from the original experiments. (ZIP)

S2 File Original data underlying Fig 4B αLRRK2 panel. (JPG)

S3 File Original data underlying Fig 4A αLRRK2 P-S935 panel. (JPG)

S4 File Data underlying the Fig 4B αIRF3 P-S396 panel. Replicate data from the time of the original experiments.
(TIF)

S5 File Data underlying the Fig 4B αp38 panel. Replicate data from the time of the original experiments.
(JPG)

S6 File Original data underlying Fig 8 Wildtype α IKKα/β P-176/180 panel. (JPG)
