# Supplementary figures and images for: The IkappaB Kinase Family Phosphorylates the Parkinson’s Disease Kinase LRRK2 at Ser935 and Ser910 during Toll-Like Receptor Signaling
Source: PLoS One. 2012 Jun 18;7(6):e39132. doi: 10.1371/journal.pone.0039132 (PMC3377608; doi:10.1371/journal.pone.0039132)

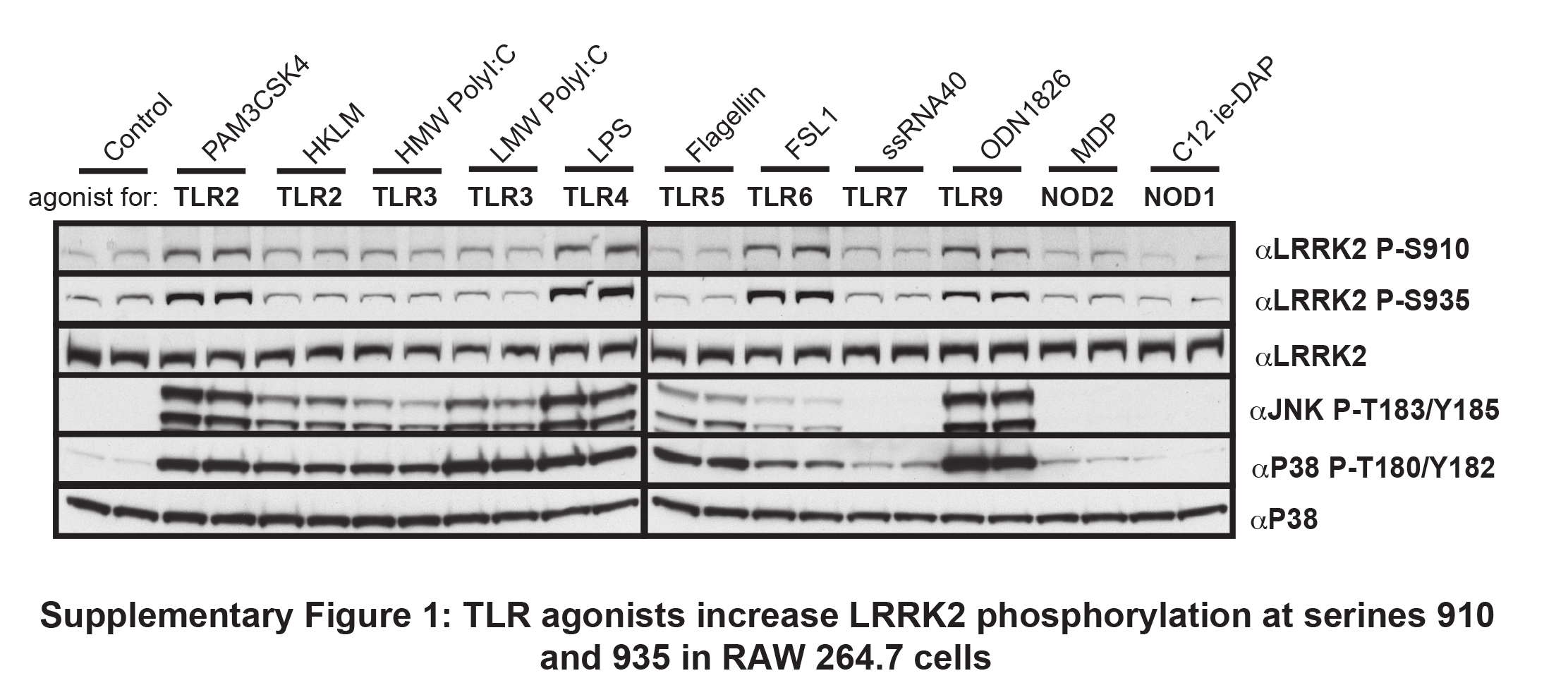

Supplement: Figure S1 — TLR agonists increase LRRK2 phosphorylation at Serines 910 and 935 in RAW 264.7 cells. RAW 264.7 macrophages were treated with the following TLR agonists for 1 h. 1 µg/ml Pam3CSK4, 108 cells HKLM, 10 µg/ml LMW and HMW Poly(I:C), 100 ng/ml LPS, 10 µg/ml Flagellin, 1 µg/ml FSL1, 1 µM CLO97 and 2.5 µM ODN1826. Lysates were subjected to immunoblot with the indicated antibodies. Results are representative of at least two independent experiments. (TIF) [file pone.0039132.s001.tif]

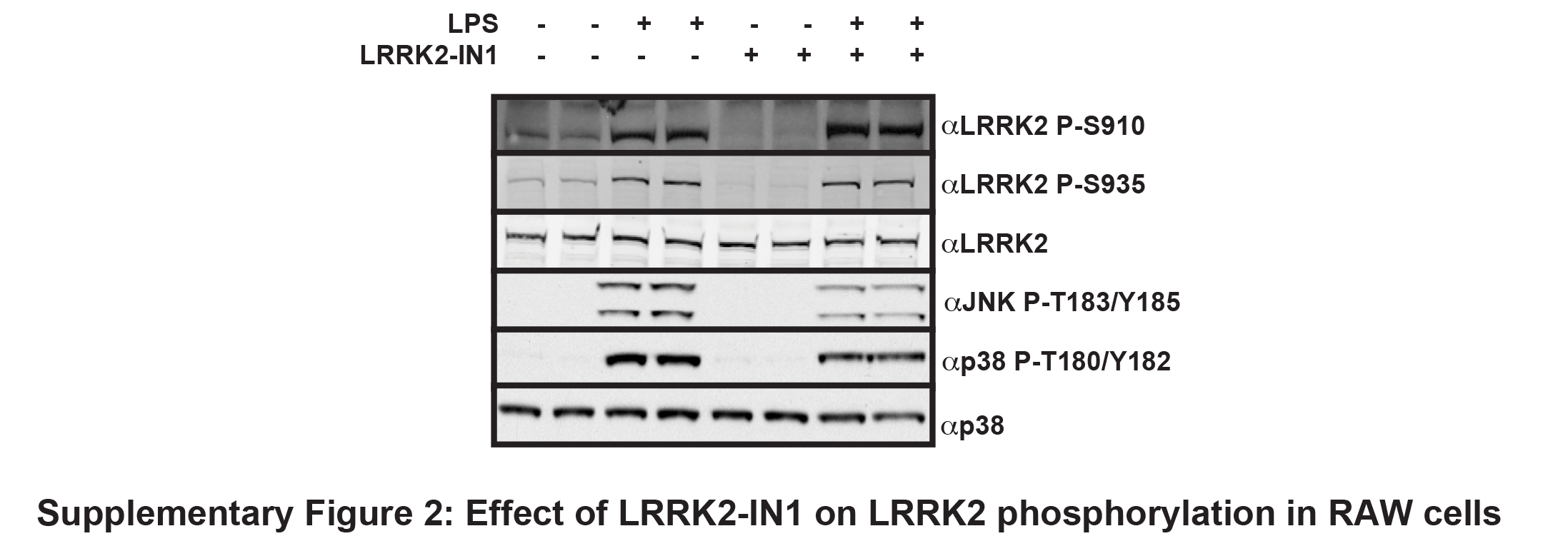

Supplement: Figure S2 — Effect of LRRK2-IN1 on LRRK2 phosphorylation in RAW cells. RAW 264.7 macrophages were pre-treated with 1 µM LRRK2-IN1 or DMSO as control for 1 h then stimulated plus or minus 100 ng/ml LPS for 1 h. Cell lysates were prepared and subjected to immunoblot with the indicated antibodies. Results are representative of at least two independent experiments. (TIF) [file pone.0039132.s002.tif]
