# Supplementary material for: The IkappaB Kinase Family Phosphorylates the Parkinson’s Disease Kinase LRRK2 at Ser935 and Ser910 during Toll-Like Receptor Signaling
Source: PLoS One. 2012 Jun 18;7(6):e39132. doi: 10.1371/journal.pone.0039132 (PMC3377608; doi:10.1371/journal.pone.0039132)
Supplement: Method S1 — Synthesis of NG25. (DOC) [file pone.0039132.s003.doc]

**Method S1. Synthesis of NG25**

Unless otherwise noted, reagents and solvents were obtained from commercial suppliers and were used without further purification. ^1^H NMR spectra were recorded on 400 MHz (Varian 7600 AS), and chemical shifts are reported in parts per million (ppm, δ) downfield from tetramethylsilane (TMS). Coupling constants (*J*) are reported in Hz. Spin multiplicities are described as s (singlet), brs (broad singlet), t (triplet), q (quartet), and m (multiplet). Mass spectra were obtained on a Waters Micromass ZQ instrument. Preparative HPLC was performed on a Waters Symmetry C18 column (19 x 50 mm, 5µM) using a gradient of 5-95% acetonitrile in water containing 0.05% trifluoacetic acid (TFA) over 8 min (10 min run time) at a flow rate of 30 mL/min. Purities of assayed compounds were in all cases greater than 95%, as determined by reverse-phase HPLC analysis.

A solution of m-chloro-perbenzoic acid (m-CPBA) (102 g, 0.457 mM) in dichloromethane (100 mL_) at 0^o^C was treated over 1 hr with a solution of 7-azaindole (**1**) (20 g, 0.1692 mM ) in dichloromethane (120 mL). The mixture was allowed to warm to 25^o^C, stirred for 2 hr., and concentrated. The resultant residue was dissolved in MeOH (200 mL) and saturated aqueous K_2_CO_3_ (50 mL), mixed for 30 min., and filtered. The filtrate was concentrated, and the resultant residue was purified by silica gel column using 10 percent MeOH/CHCI_3_ as eluting solvent. The pure fractions were combined and concentrated to provide 20g (85%) of 7-azaindole-7-oxide (**2**) as pale brown solid.

To a stirred and ice-cooled solution of compound **2** (6.3 g, 0.047 mol) in 50 mL of trifluoroacetic acid was added 22 mL of fuming nitric acid. As soon as addition of the nitric acid was completed, the mixture was rapidly poured into ice, and 110 mL of 12 N NaOH was added dropwise to obtain a yellow precipitate. The reaction mixture was allowed to stand for 30 min at room temperature, and then the solid was filtered, washed with cold water, and dried in vacuo to yield 6.5 g of a mixture of **3** and **4*.*** Recrystallization from water gave 5 g (60%) of compound **4**.

The physical data of **4** is identical as reported.

Under an argon atmosphere, 5.00 g (27.9 mmol) of 4-Nitro-1H-pyrrolo[2,3-b]pyridine 7-oxide (**4**) and 11.8 mL (55.8 mmol) of hexamethyldisilazane are initially charged in 290 mL of THF. At room temperature, 10.8 mL (140 mmol) of methyl chloroformate are added. The solution is stirred at room temperature overnight. The reaction solution is filtered through a silica gel cartridge and the cartridge is washed with dichloromethane/methanol 10:1. 2.8 g (70%) of **6** was obtained.

To a stirred and ice-cooled solution of compound **5** (1 g, 5 mmol) and SEMCl (2.9 g, 7.5 mmol) in 25 mL of THF was slowly added NaH (60%, 300 mg, 7.5 mmol). The reaction mixture was allowed to stand for 30 min at 0 ^o^C, and then warmed to RT. After 3 hr saturated NH_4_Cl solution was added. The mixture was extracted with ethyl acetate, the organic phase was concentrated and purified with column chromatography (hexane : ethyl acetate 5:1). 1.21 g (74%) of **6** was obtained. ^1^H NMR (400 MHz, CDCl_3_) δ 7.95 (s, 1H), 7.63 (d, *J* = 3.6 Hz, 1H), 7.19 (d, *J* = 3.2, 1H), 5.70 (s, 2H), 3.55 (t, *J* = 8.0 Hz, 2H), 0.93 (t, *J* = 8.0 Hz, 2H), -0.05 (s, 9H). MS (ESI) *m/z* 328 (M+H)^+^.

To a stirred solution of compound **6** (570 mg, 2 mmol) and **7** (340 mg, 2.2 mmol) in 13 mL of DMSO was added K_2_CO_3_ (830 mg, 6 mmol). The reaction mixture was allowed to stand for 5 hr at 100 ^o^C, and then cooled to RT. The mixture was acidified with 1N HCl solution and extracted with ethyl acetate, the organic phase was concentrated and purified with column chromatography (dichloromethane : methanol 20:1). 690 mg (80%) of **8** was obtained.

Pd/C (5%, 120 mg) was added to a stirred solution of compound **8** (690 mg, 1.6 mmol) in 10 mL of methanol, the mixture was stirred under hydrogen atmosphere for 2 or 3 days. The reaction was monitored by TLC, more Pd/C might be needed for complete conversion during the period. Then the reaction solution is filtered through a silica gel cartridge and the cartridge is washed with dichloromethane/methanol 20:1. 510 mg (80%) of **9** was obtained. ^1^H NMR (400 MHz, CDCl_3_) δ 8.30 (d, *J* = 5.6 Hz, 1H), 8.02 (d, *J* = 8.0 Hz, 1H), 7.90 (s, 1H), 7.48 (d, *J* = 8.0 Hz, 1H), 7.33 (d, *J* = 3.6 Hz, 1H), 6.52 (d, *J* = 3.6, 1H), 6.44 (d, *J* = 5.6, 1H), 5.78 (s, 2H), 3.65 (t, *J* = 8.0 Hz, 2H), 2.37 (s, 3H), 0.99 (t, *J* = 8.0 Hz, 2H), 0.00 (s, 9H). MS (ESI) *m/z* 399 (M+H)^+^.

To a stirred solution of compound **9** (400 mg, 1 mmol) and **10** (430 mg, 1.5 mmol) in 10 mL of dichloromethane was added HATU (570 mg, 1.5 mmol), DMAP (185mg, 1.5 mmol) and DIEA(520 μl, 3 mmol). The reaction mixture was allowed to stand for 24 hr at room temperature, then eluted with ethyl acetate and washed with water, the organic phase was concentrated and purified with column chromatography (dichloromethane : methanol 15:1). 600 mg (90%) of **11** was obtained. ^1^H NMR (400 MHz, CDCl_3_) δ 8.21 (s, 1H), 8.12 (d, *J* = 5.6 Hz, 1H), 7.91 (d, *J* = 8.0 Hz, 1H), 7.74-7.68 (m, 2H), 7.69 (s, 1H), 7.41 (d, *J* = 8.0 Hz, 1H), 7.22 (d, *J* = 3.6, 1H), 6.37 (d, *J* = 3.6, 1H), 6.33 (d, *J* = 5.6, 1H), 5.64 (s, 2H), 3.61 (s, 2H), 3.55 (t, *J* = 8.0 Hz, 2H), 2.65-2.40 (m, 8H), 2.50 (q, *J* = 6.8 Hz, 2H), 2.28 (s, 3H), 1.24 (t, *J* = 6.8 Hz, 3H), 0.90 (t, *J* = 8.0 Hz, 2H), -0.08 (s, 9H). MS (ESI) *m/z* 668 (M+H)^+^.

To a stirred ice-cooled solution of compound **11** (330 mg, 0.5 mmol) in 5 mL of dichloromethane was added 1mL of TFA. The reaction mixture was allowed to stand for 30 min at 0 ^o^C, and then warmed to RT. After 5 hr the mixture was concentrated and dried by vacuum, then dissolved in 5 mL of THF, and 5 mL of 1N NaOH water solution was added. The mixture was stirred for 24 hr and extracted with ethyl acetate. The organic phase was concentrated and then recrystallized from ethyl acetate gave 188 mg (70%) of NG25**.** ^1^H NMR (400 MHz, DMSO) δ 11.77(br, 1H), 8.15 (d, *J* = 2.0 Hz, 1H), 8.08 (d, *J* = 6.0 Hz, 1H), 8.01 (dd, *J* = 8.0, 2.0 Hz, 1H), 7.88 (dd, *J* = 8.0, 1.6 Hz, 1H), 7.77 (d, *J* = 3.0 Hz, 1H), 7.68 (d, *J* = 8.4 Hz, 1H), 7.57 (d, *J* = 8.4 Hz, 1H), 7.37 (dd, *J* = 3.6, 2.8, 1H), 6.32 (d, *J* = 5.2, 1H), 6.21 (dd, *J* = 3.6, 2.0 Hz, 1H), 3.56 (s, 2H), 2.52-2.30 (m, 8H), 2.50 (q, *J* = 7.2 Hz, 2H), 2.24 (s, 3H), 1.00 (t, *J* = 7.2 Hz, 3H). MS (ESI) *m/z* 538 (M+H)^+^.
