# Supplementary material for: The IkappaB Kinase Family Phosphorylates the Parkinson’s Disease Kinase LRRK2 at Ser935 and Ser910 during Toll-Like Receptor Signaling
Source: PLoS One. 2012 Jun 18;7(6):e39132. doi: 10.1371/journal.pone.0039132 (PMC3377608; doi:10.1371/journal.pone.0039132)
Supplement: Table S4 — qRT-PCR primers. Sense and anti-sense primer sequences for the analysis of inflammatory gene expression by qRT-PCR are listed. (XLSX) [file pone.0039132.s007.xlsx]

| **Gene** | **Sense** | **Antisense** |
| --- | --- | --- |
| TNFα | CAGACCCTCACACTCAGATCATC | GGCTACAGGCTTGTCACTCG |
| IL-6 | TTCCATCCAGTTGCCTTCTTG | AGGTCTGTTGGGAGTGGTATC |
| IL-12(p35) | TATCTCTATGGTCAGCGTTCC | TGGTCTTCAGCAGGTTTCG |
| IL-12(p40) | TCATCAGGGACATCATCAAACC | TGAGGGAGAAGTAGGAATGGG |
| IFNb | GGAAAAGCAAGAGGAAAGATTGAC | CCACCATCCAGGCGTAGC |
| IL-1b | GACGGACCCCAAAAGATGAAGG | GTGATACTGCCTGCCTGAAGC |
| GAPDH | GCCTTCCGTGTTCCTACCC | TGCCTGCTTCACCACCTTC |

**Supplementary table 1: qRT-PCR primers**
