# Supplementary figures and images for: Expression of Concern: The IkappaB Kinase Family Phosphorylates the Parkinson’s Disease Kinase LRRK2 at Ser935 and Ser910 during Toll-Like Receptor Signaling
Source: PLoS One. 2025 Aug 26;20(8):e0330958. doi: 10.1371/journal.pone.0330958 (PMC12380296; doi:10.1371/journal.pone.0330958)

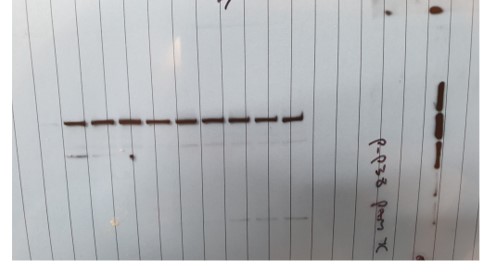

Supplement: S1 File — (ZIP) [file pone.0330958.s001.zip › S1 File/Total LRRK2 myd KO _high exposure.jpg]

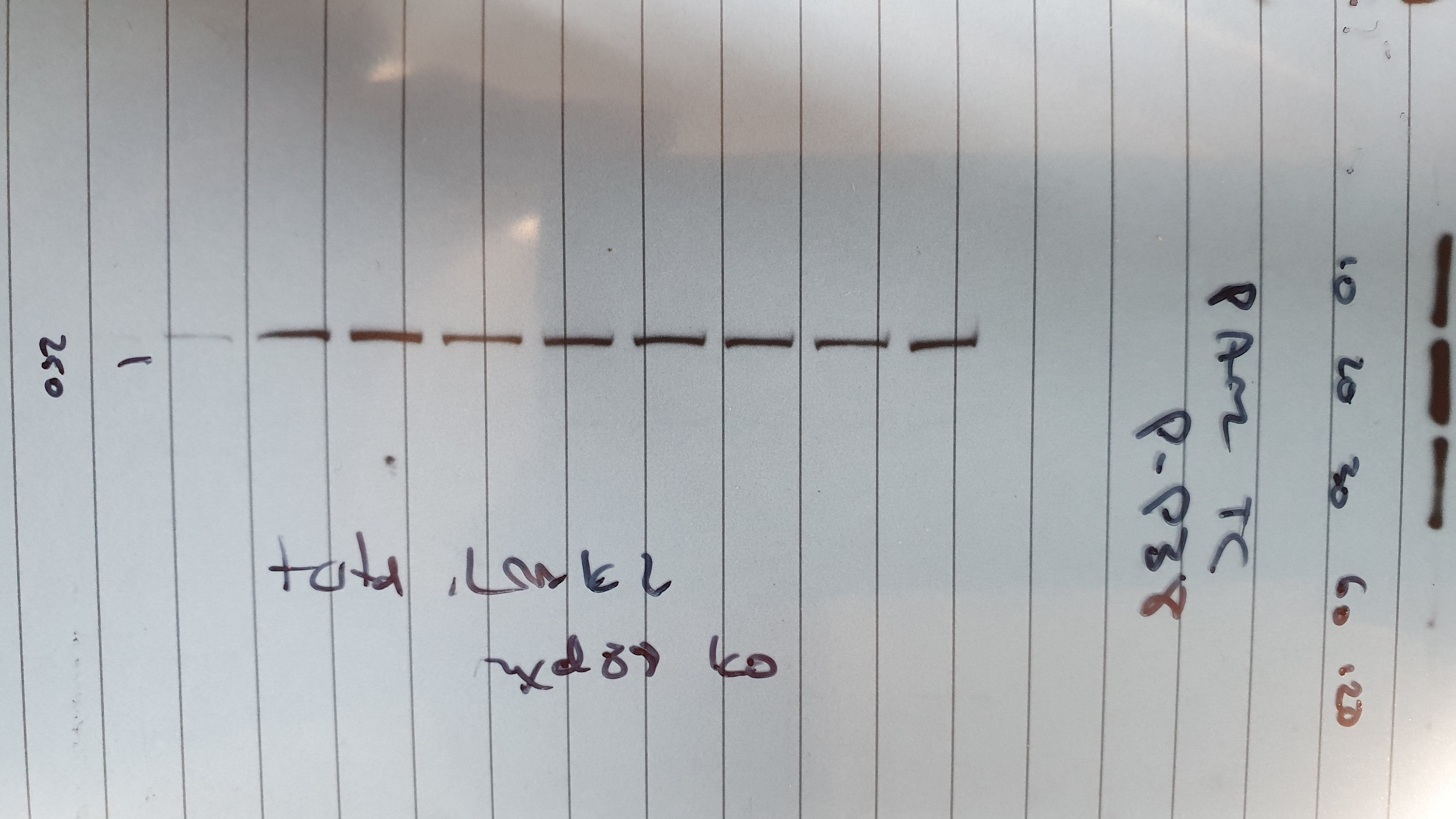

Supplement: S1 File — (ZIP) [file pone.0330958.s001.zip › S1 File/Total LRRK2 myd KO _low exposure.jpg]

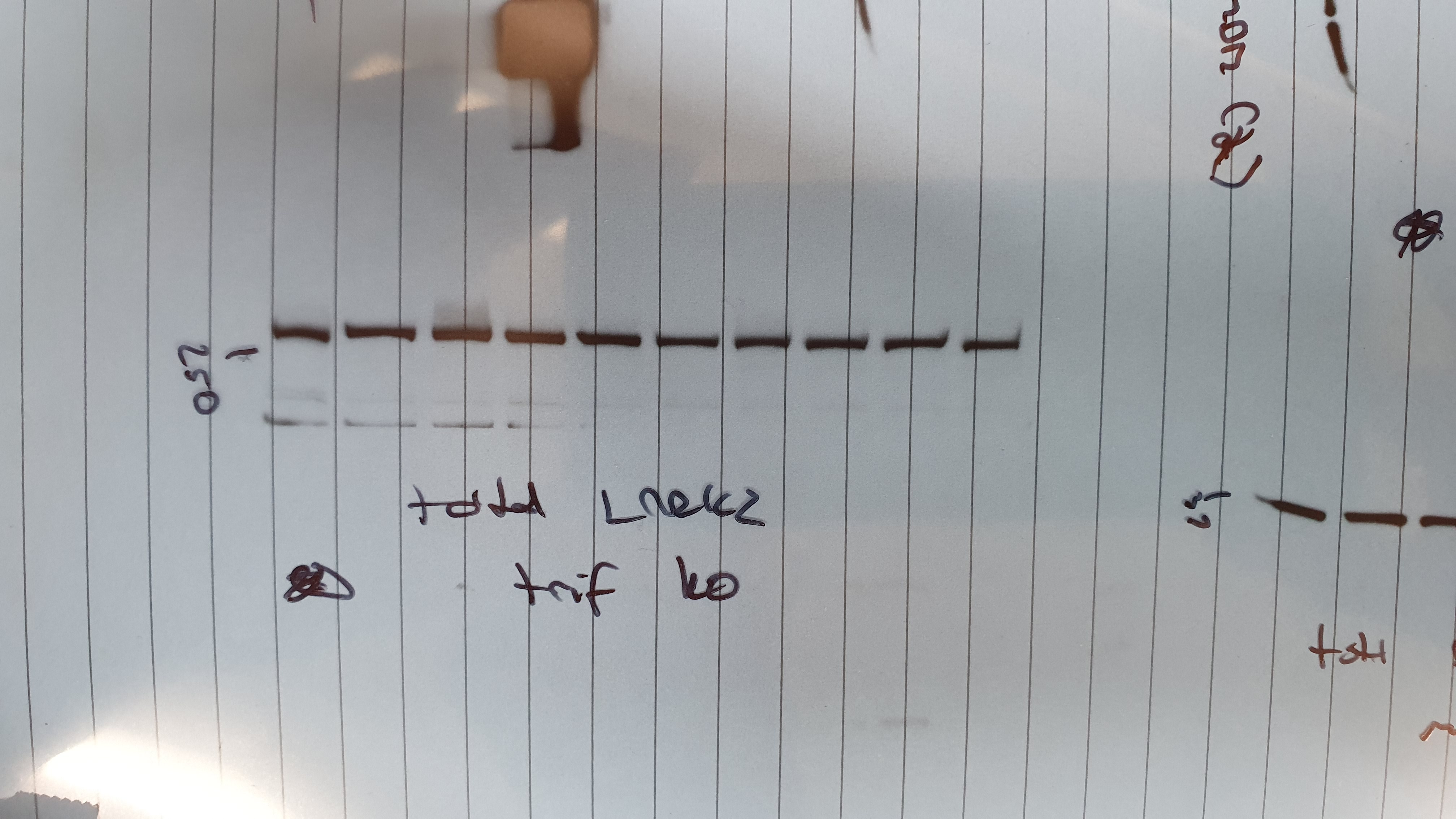

Supplement: S2 File — (JPG) [file pone.0330958.s002.jpg]

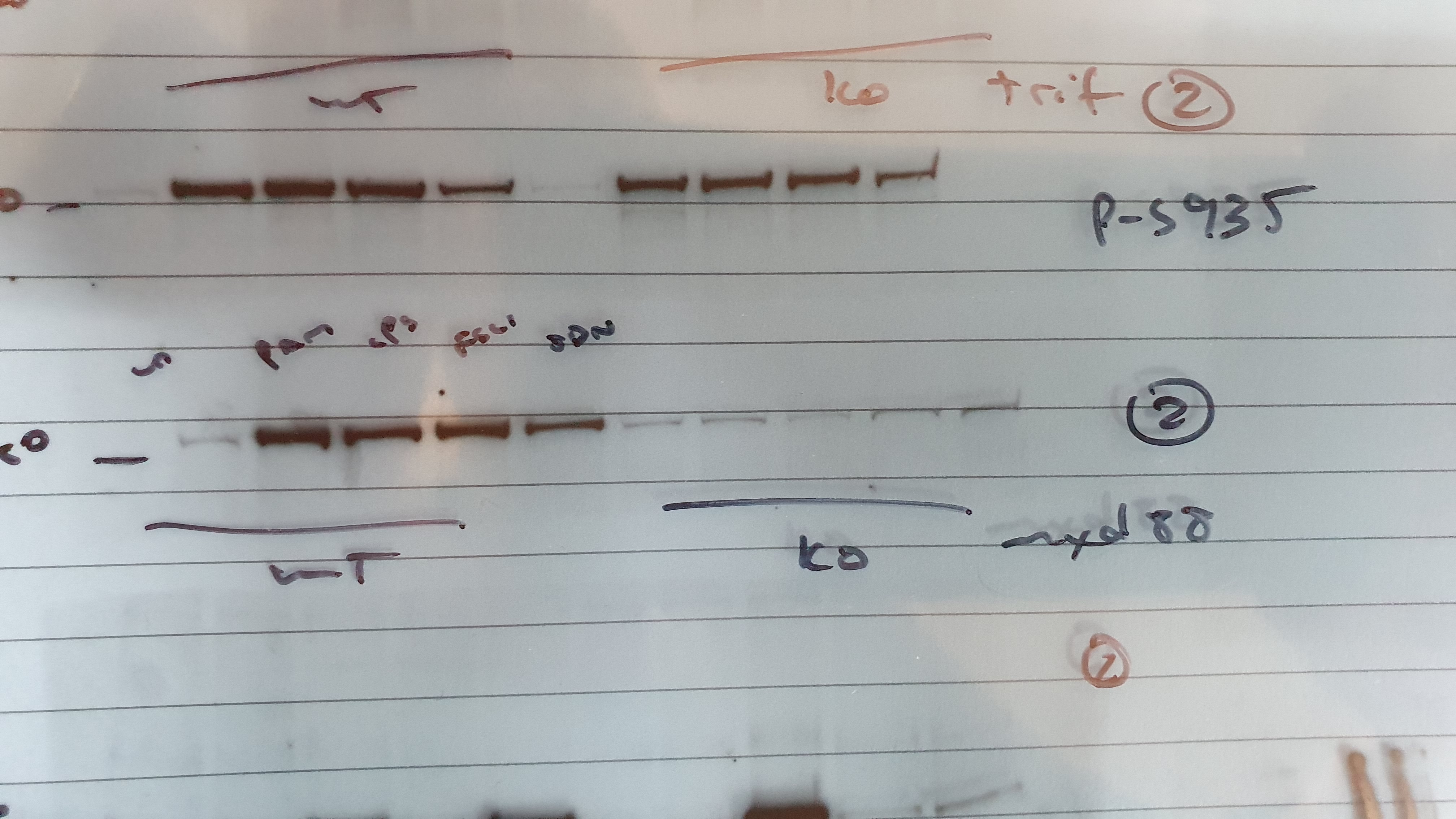

Supplement: S3 File — (JPG) [file pone.0330958.s003.jpg]

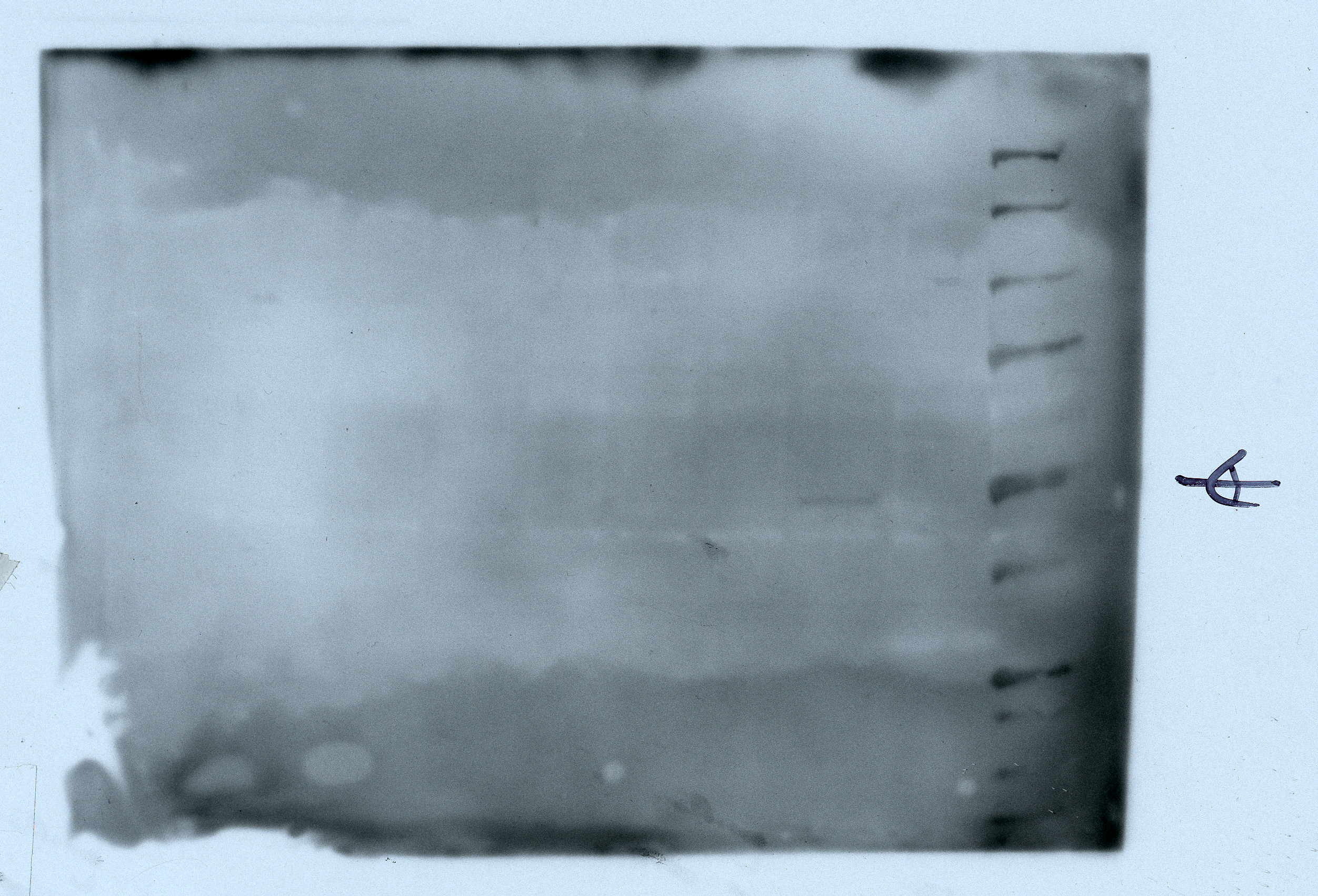

Supplement: S4 File — Replicate data from the time of the original experiments. (TIF) [file pone.0330958.s004.tif]

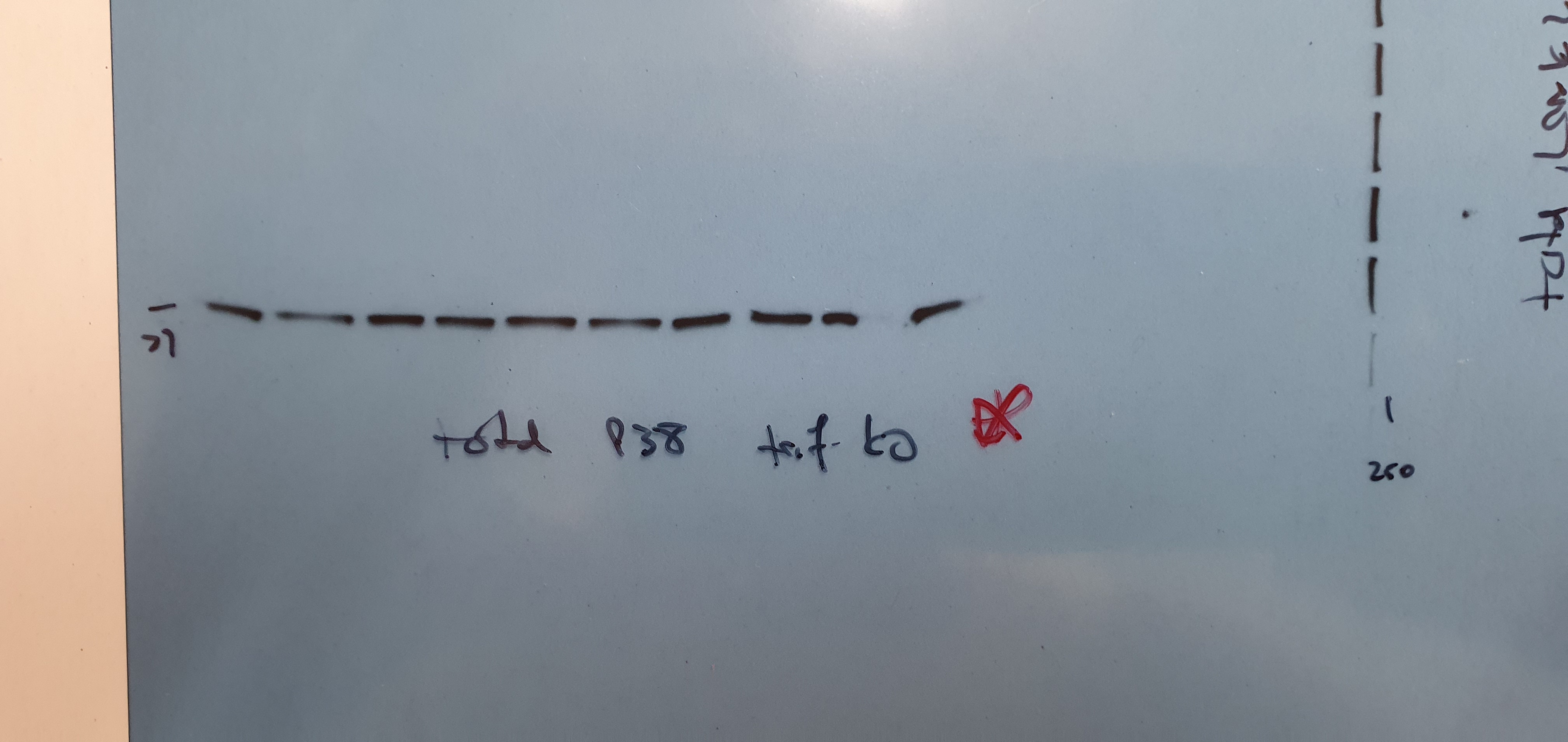

Supplement: S5 File — Replicate data from the time of the original experiments. (JPG) [file pone.0330958.s005.jpg]

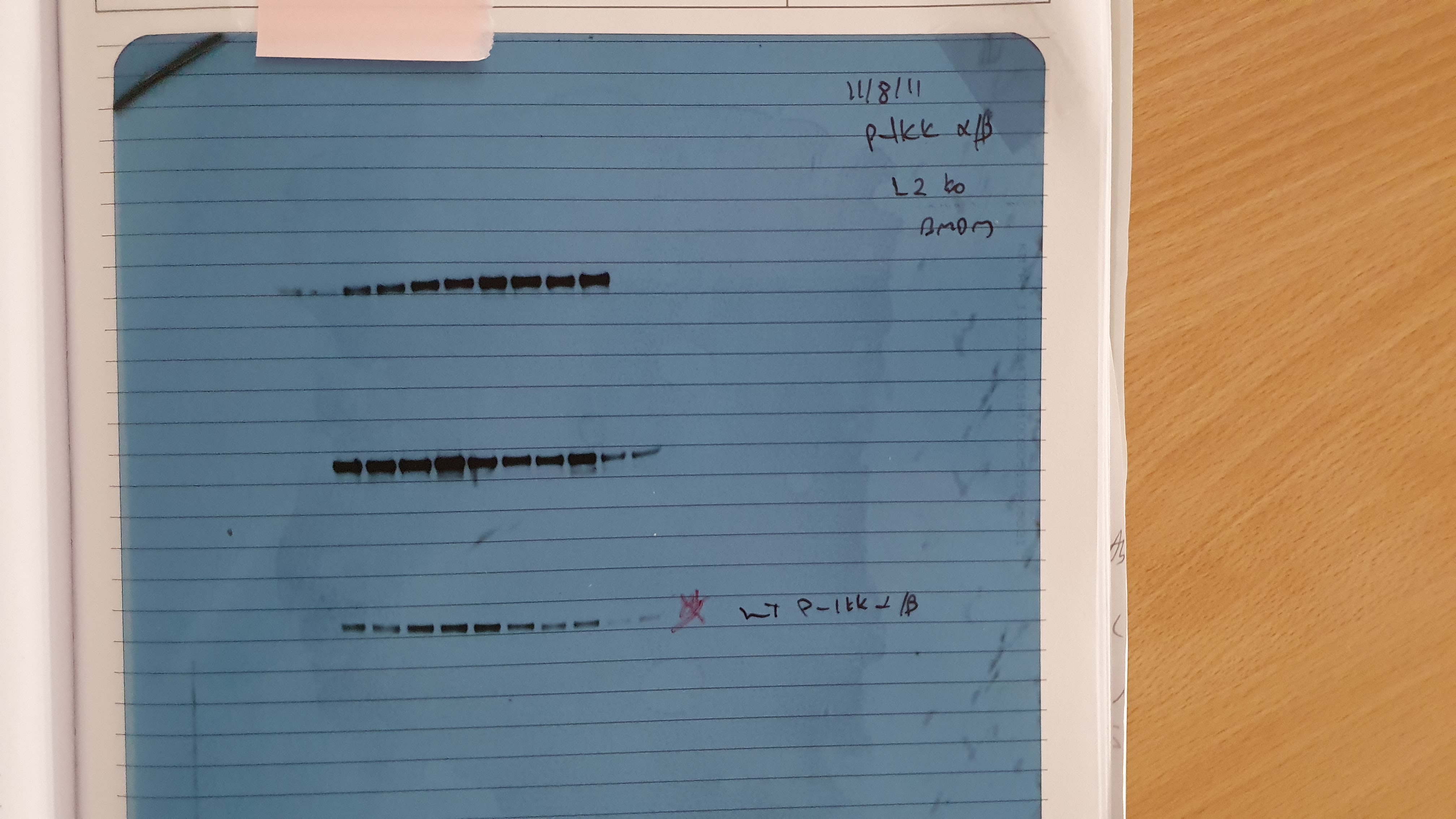

Supplement: S6 File — (JPG) [file pone.0330958.s006.jpg]
